# Abnormal bleeding in the cardiac operating room: An observational study of interrater reliability between anesthetists and surgeons

**DOI:** 10.1016/j.xjon.2026.101716

**Published:** 2026-03-06

**Authors:** Alexandre Sebestyen, Alexandre Behouche, Julien Picard, Joris Giai, Corentin Leroy, Jean-Noel Evain, Olivier Chavanon, Pierre Albaladejo

**Affiliations:** a Department of Cardiac Surgery, Grenoble Alpes University Hospital, Grenoble, France; b Alps Research Assessment and Simulation Centre (CESAR), ThEMAS Group, TIMC Laboratory, UMR CNRS 5525, Grenoble Alpes University, Grenoble, France; c Department of Anesthesia and Intensive Care, Grenoble Alpes University Hospital, Grenoble, France; d Deparment of Public Health and Biostatistics Department, INSERM CIC 1406, Grenoble Alpes University Hospital, Grenoble, France

**Keywords:** cardiac surgery, intraoperative hemorrhage, blood transfusion, situational awareness, interrater reliability

## Abstract

**Objectives:**

Postcardiopulmonary bypass bleeding is frequent and distinctive because of its associated coagulopathy. Despite diagnostic and therapeutic progress, substantial variability persists in hemostatic transfusion practices. Because transfusion of hemostatic components should target abnormal bleeding, this study assessed the situational awareness of anesthetists and surgeons in such situations.

**Methods:**

A nationwide questionnaire using real intraoperative video clips—3 normal, 8 ambiguously abnormal, and 6 clearly abnormal situations—evaluated interrater reliability for identifying abnormal bleeding, inferring residual coagulopathy, and deciding on immediate hemostatic transfusion. Agreement was measured by Gwet's AC1 (γ [95% CI]) and classified per Landis and Koch. A generalized mixed linear model assessed the impact of abnormality level on intra-dyad agreement.

**Results:**

Among 47 surgeons and 54 anesthetists, interrater reliability for abnormal bleeding perception was “moderate” (γ = 0.47 [0.32-0.62]) but significantly lower for abnormal situations (γ = 0.43 [0.28-0.58] vs γ = 0.96 [0.91-1.00]), and “poor” for ambiguously abnormal ones (γ = 0.20 [0.03-0.37]). Reliability for identifying residual coagulopathy remained “poor” overall (γ = 0.27 [–0.28 to 0.58]). For clearly abnormal situations, agreement on immediate hemostatic transfusion was “moderate” (γ = 0.51 [0.44-0.58]) but differed by specialty (γ = 0.38 [0.28-0.49] for anesthetists vs γ = 0.65 [0.52-0.78] for surgeons), and intra-dyad agreement was 6 times lower than for ambiguously abnormal situations (odds ratio, 0.18 [0.17-0.19]).

**Conclusions:**

Abnormal bleeding during cardiac surgery is complex and often difficult to assess instantly. Beyond cognitive aids, enhanced training to improve shared situational awareness and develop a common mental model could reduce practice variability and strengthen patient blood management and safety.

## Graphical Abstract

French cardiac anesthetists (n = 54) and surgeons (n = 47) were asked on a panel of different real-time post-CPB video recordings (N = 17) whether (1) the bleeding was “abnormal”, (2) they suspected a residual coagulopathy, and (3) there was a need for immediate hemostatic transfusion. Interrater reliability—calculated by Gwet's AC1—in the face of abnormal situations was insufficient (<0.80) but better between surgeons than between anesthetists on hemostatic transfusion in the face of clearly abnormal bleeding situations, with 6 times lower risk of agreement. This highlighted the complexity of the post-CPB bleeding management, and possible variation in hemostatic transfusion practices could be attributable to the low level of situational awareness. Enhancing nontechnical skills by specific tools and training to optimize intraoperative bleeding management appears crucial. *CPB*, Cardiopulmonary bypass; *B0*, normal situation; *B?*, ambiguously abnormal bleeding situation; *B!*, clearly abnormal bleeding situation; *B+*, abnormal bleeding situations; including B? ones and B! ones.

**Figure:**
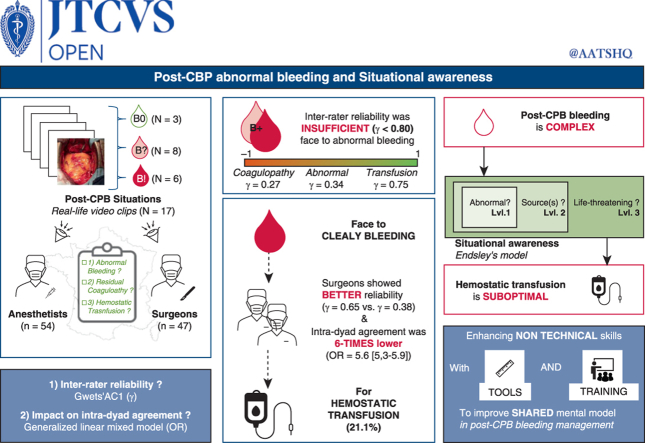

**Figure:**
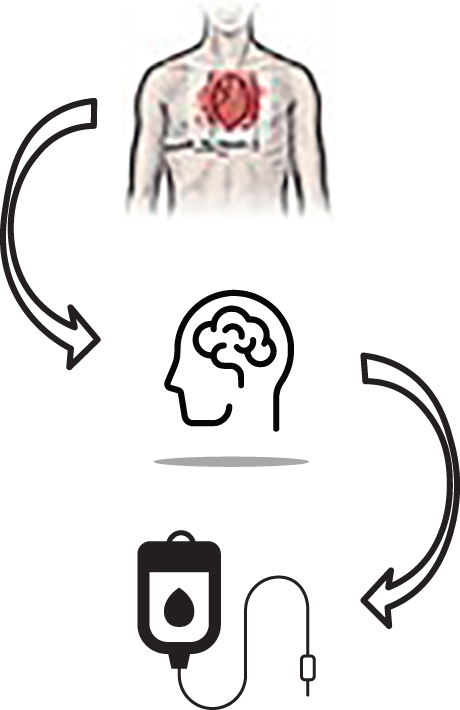
Situational awareness, from abnormal bleeding to hemostatic transfusion.

**Central Message**

Interrater reliability may be insufficient in the face of the complexity of abnormal bleeding in cardiac surgery, with a risk of hemostatic transfusion from a simple check at a glance.

**Perspective**

Enhancing shared situational awareness between surgeons and anesthetists, by using cognitive tools and specific training, may optimize postcardiopulmonary bypass bleeding management.

Bleeding is common in open surgery and correlates with operative morbidity.^1^^,^^2^ Preserving blood resources is essential for patient safety, and both surgeons and anesthetists are trained to achieve effective hemostasis. Hemostatic transfusion plays a key role in managing coagulopathy and is commonly used in cardiac surgery because of procedural and patient-specific factors.^3^, ^4^, ^5^

Although hemostatic blood components are effective, their use carries risks.^2^^,^^6^, ^7^, ^8^ Despite existing recommendations, evidence remains limited for the intraoperative setting, and transfusion exposure still varies widely.^9^ Viscoelastic testing–guided algorithms have reduced, but not eliminated, inappropriate transfusion.^10^^,^^11^ Except a few evidence-based indications for prophylactic use, blood products should be administered mainly in response to abnormal bleeding.

Optimizing nontechnical skills, particularly situational awareness, is essential to improve safety and decision-making in the operating theater.^12^, ^13^, ^14^ Despite advances in the assessment of coagulopathy,^7^^,^^15^ hemostatic transfusion practices remain heterogeneous, likely as a result of inconsistent definitions of abnormal bleeding. This study therefore aimed to evaluate interrater reliability between anesthetists and surgeons when facing various intraoperative postcardiopulmonary bypass (CPB) bleeding scenarios.

## Methods

### Ethics Statement

The study protocol was reviewed and approved by the Clinical Research and Innovation Department of Grenoble-Alpes University Hospital and by the Scientific Committee of the French Society of Anesthesiology and Intensive Care (institutional review board no. 00010254-2025-103) on July 4, 2025. Completion of the questionnaire implied informed consent from respondent for the publication of study data, and no personally identifiable information was collected.

### Respondents and Experimental Simulations

A questionnaire was distributed to all cardiac surgeons and cardiothoracic anesthetists registered with the French Society of Thoracic and Cardiovascular Surgery and the Heart-Thorax-Vessels Anesthesiology Section of the French Society of Anesthesiology and Intensive Care. The survey was hosted on SurveyMonkey (SurveyMonkey Inc), with video sequences accessible via Vimeo through restricted links.

Respondents first provided basic information regarding their professional status: length of practice (<10 or ≥10 years), individual annual volume of major cardiac procedures performed, including on-pump and off-pump coronary artery bypass grafting (<100, 100-200, or >200 procedures per year), and center affiliation (public or private).

They then viewed a randomized panel of video clips depicting post-CPB operative fields 10 to 20 minutes after previously planned hemostatic therapy was done. Each video clips lasted about 15 seconds (between 10 and 20 seconds), simulating an observation at a glance (Videos 1-17). This panel was arbitrarily selected and divided into 3 predefined types of post-CPB bleeding situations: “normal” (B0, N = 3) and “abnormal” (B+, N = 14), the latter subdivided into “ambiguously abnormal” (B?, N = 8) and “clearly abnormal” (B!, N = 6). Appendix E1 details how we defined the different situations. For each clip, respondents judged whether the bleeding appeared abnormal, whether residual coagulopathy was present, and whether immediate hemostatic transfusion was indicated before any additional testing. Clinical context was intentionally excluded, except for the timing of recording (10-20 minutes after weaning from CPB) and the administration of previously planned reversal therapy. For all cases, temperature >36 °C, pH >7.2, and ionized calcium >1.0 mmol/L were assumed. Some procedural clues may be visible in the operative field, and their interpretation was left to the respondents’ discretion. Appendix E2 provides a shortened English translation of the questionnaire. Video clips are presented one by one in the individual videos, and Vimeo links are also provided in Appendix E1 and E2.

### Statistical Analysis

Only fully completed, questionnaires were analyzed. Respondent characteristics were summarized as counts (%) and compared between surgeons and anesthetists using the Fisher exact test (*P* < .05), with Bonferroni correction when appropriate.

After we determined the prevalence of each response across the entire panel and for each bleeding category, interrater reliability was assessed using Gwet's AC1 coefficient (γ) with 95% CIs, both overall and by each characteristics (specialty, length of practice, individual annual volume of major cardiac procedures, and center affiliation). Reliability was classified as “excellent” (γ > 0.80), “good” (γ = 0.60-0.80), “moderate” (γ = 0.40-0.60), or “poor” (γ < 0.40). Significant differences were defined by nonoverlapping CIs.

Using a generalized linear mixed-effects model fitted by maximum likelihood estimation, we calculated odds ratios (ORs) with 95% CIs to evaluate the influence of bleeding situation type on intra-dyad agreement, or the same judgment or decision between a pair comprising 1 surgeon and 1 anesthetist, regarding the perception of bleeding, the supposition of residual coagulopathy, and the decision on immediate hemostatic transfusion. Comparisons were made between abnormal (B+) and normal (B0) situations, and between clearly abnormal (B!) and ambiguously abnormal (B?) situations. All analyses were performed using R software (R Core Team, 2024) with irrCAC (v1.0; Gwet, 2019) and glmm (v1.4.5; Kurdson, Geyer, Benson; 2024) packages.

## Results

### Respondent Characteristics

After we excluded 58 incomplete questionnaires, we analyzed 101 fully completed ones from 47 surgeons and 54 anesthetists, corresponding to 13.6% and 6.6% of overall French cardiac physicians, respectively (Table 1). Most respondents worked in public centers. Despite a comparable length of experience, surgeons reported a greater annual volume of major cardiac procedures.

**Table 1:** Respondents’ characteristics

Characteristics	Total respondents (n = 101)	Surgeons (n = 47)	Anesthetists (n = 54)	*P* value
Experience <10 y	56 (55.4)	26 (55.3)	30 (55.6)	1.000
Annual major cardiac procedures				<.001
<100	29 (28.7)	2 (4.3)	27 (50.0)	
100-200	35 (34.7)	17 (36.2)	18 (33.3)	
>200	37 (36.6)	28 (59.6)	9 (16.7)	
Public affiliation center	84 (83.2)	36 (76.6)	48 (88.9)	.116

### Interrater Reliability

**Figure 1:**
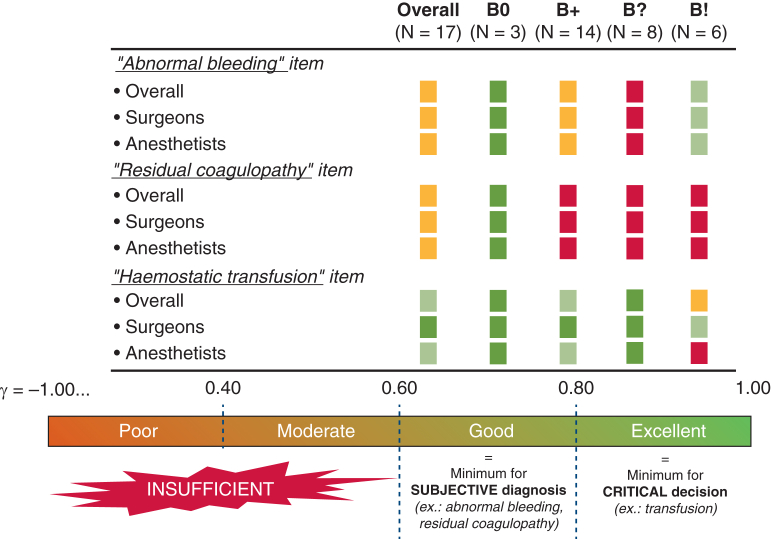
Graphical table of interrater reliability. The panel of situations was divided in 3 “normal” (B0) ones and 14 “abnormal” (B+) ones, including 8 “ambiguously” (B?) and 6 “clearly” (B!). The qualitative classification of interrater reliability, calculated by Gwet's AC1 test (γ) (available in Table 2), followed the definitions of Landis and Koch. Interpretation in clinical practice is summarized in the Figure.

The overall prevalence of “abnormal bleeding,” “residual coagulopathy,” and “immediate hemostatic transfusion” items were 55.7%, 27.5%, and 9.9%, respectively. The interrater reliability was “moderate” for “abnormal” bleeding” and “residual coagulopathy” items, γ = 0.47 [0.32-0.62] and γ = 0.43 [0.32-0.54], respectively (Table 2 and Figure 1).

**Table 2:** Detailed prevalence, observed agreement, and interrater reliability

Responses	Overall (N = 17)			B0 (N = 3)			B+ (N = 14)			B? (N = 8)			B! (N = 6)		
	f	Po	γ[95% CI]	f	Po	γ[95% CI]	f	Po	γ[95% CI]	f	Po	γ[95CI]	f	Po	γ[95% CI]
"Abnormal bleeding" item															
Overall (n = 101)	55.7	73.2	0.47 [0.32-0.62]	2.0	96.1	0.96 [0.91-1.00]	67.3	68.2	0.43 [0.28-0.58]∗	52.0	59.9	0.20 [0.03-0.37]	87.6	79.3	0.74 [0.67-0.81]†
Surgeons (n = 47)	52.7	76.0	0.52 [0.33-0.71]	0.0	1.00	1.00 [1.00-1.00]	64.0	79.8	0.45 [0.28-0.64]∗	45.7	63.5	0.28 [0.02-0.53]	88.3	80.6	0.76 [0.61-0.90]†
Anesthetists (n = 54)	58.4	71.7	0.45 [0.38-0.61]	3.7	92.9	0.92 [0.84-1.00]	70.1	67.1	0.43 [0.29-0.58]∗	57.4	58.3	0.18 [0.03-0.34]	87.0	78.9	0.73 [0.58-0.88]†
Experience ≥10 y (n = 45)	50.7	70.9	0.42 [0.25-0.59]	0.7	98.5	0.98 [0.96-1.00]	61.4	64.9	0.33 [0.20-0.46]∗	45.6	59.6	0.20 [0.03-0.37]	82.6	72.0	0.61 [0.48-0.73]†
Experience <10 y (n = 56)	59.6	75.7	0.53 [0.36-0.71]	3.7	94.2	0.94 [0.88-1.00]	71.6	71.7	0.53 [0.36-0.69]∗	56.9	61.3	0.24 [0.04-0.45]	91.1	85.6	0.83 [0.71-0.95]†
Activity <100/y (n = 29)	61.0	71.1	0.49 [0.29-0.61]	6.9	87.4	0.85 [0.69-1.00]	72.7	67.6	0.46 [0.31-0.61]∗	61.2	57.8	0.20 [0.08-0.31]	87.9	80.5	0.75 [0.57-0.93]†
Activity 100-200/y (n = 37)	56.0	76.8	0.54 [0.35-0.74]	0.0	1.00	1.00 [1.00-1.00]	68.0	71.9	0.50 [0.32-0.68]∗	50.7	62.7	0.25 [-0.01-0.52]	91.0	84.2	0.81 [0.70-0.92]†
Activity >200/y (n = 35)	51.1	72.3	0.45 [0.26-0.63]	0.0	1.00	1.00 [1.00-1.00]	62.0	66.4	0.36 [0.21-0.52]∗	45.7	61.0	0.23 [0.01-0.44]	83.8	73.5	0.64 [0.50-0.77]†
Public center (n = 84)	56.7	72.7	0.46 [0.29-0.63]	2.4	95.4	0.95 [0.90-1.00]	62.3	67.8	0.43 [0.28-0.58]∗	54.2	59.5	0.20 [0.02-0.38]	87.1	78.9	0.73 [0.59-0.86]†
Private center (n = 17)	51.2	76.4	0.53 [0.35-0.71]	0.0	1.00	1.00 [1.00-1.00]	62.2	71.3	0.46 [0.29-0.63]∗	41.2	63.6	0.29 [0.05-0.54]	90.2	81.6	0.78 [0.70-0.85]†
"Residual coagulopathy" item															
Overall (n = 101)	27.5	65.7	0.43 [0.32-0.54]	1.7	96.8	0.97 [0.93-1.00]	33.0	59.1	0.27 [0.16-0.37]∗	29.8	61.6	0.34 [0.19-0.49]	37.3	55.8	0.17 [0.11-0.23]
Surgeons (n = 47)	26.8	67.0	0.46 [0.29-0.62]	0.0	1.00	1.00 [1.00-1.00]	32.5	59.9	0.29 [0.14-0.53]∗	28.7	64.1	0.39 [0.17-0.61]	37.6	54.4	0.14 [0.06-0.22]
Anesthetists (n = 54)	28.1	64.7	0.41 [0.28-0.54]	3.1	94.0	0.94 [0.87-1.00]	33.5	58.5	0.25 [0.16-0.34]∗	30.8	60.0	0.30 [0.17-0.43]	37.0	56.5	0.18 [0.08-0.29]
Experience ≥10 y (n = 45)	26.8	67.2	0.47 [0.32-0.62]	0.7	98.5	0.98 [0.96-1.00]	32.4	60.5	0.31 [0.18-0.43]∗	26.9	65.5	0.45 [0.26-0.63]	39.6	53.9	0.12 [0.05-0.18]†
Experience <10 y (n = 56)	28.1	65.5	0.42 [0.28-0.56]	3.0	95.3	0.95 [0.90-1.00]	33.5	59.1	0.26 [0.16-0.37]∗	31.3	60.6	0.30 [0.13-0.47]	36.3	57.1	0.21 [0.10-0.32]
Activity <100/y (n = 29)	30.0	62.5	0.35 [0.23-0.48]	5.7	89.2	0.88 [0.75-1.00]	35.2	56.8	0.21 [0.12-0.29]∗	32.3	58.3	0.26 [0.13-0.39]	39.1	54.9	0.14 [0.03-0.25]
Activity 100-200/y (n = 37)	27.3	67.4	0.46 [0.30-0.61]	0.0	1.00	1.00 [1.00-1.00]	33.2	60.3	0.29 [0.17-0.41]∗	30.7	62.6	0.35 [0.16-0.54]	36.5	57.4	0.21 [0.07-0.34]
Activity >200/y (n = 35)	25.5	66.5	0.46 [0.31-0.61]	0.0	1.00	1.00 [1.00-1.00]	31.0	59.3	0.29 [0.17-0.41]∗	26.8	63.3	0.40 [0.22-0.58]	36.7	54.0	0.14 [0.06-0.23]
Public center (n = 84)	27.9	65.2	0.42 [0.28-0.56]	2.0	96.1	0.96 [0.92-1.00]	33.4	58.6	0.25 [0.16-0.35]∗	30.7	60.6	0.31 [0.16-0.47]	37.1	55.9	0.17 [0.09-0.25]
Private center (n = 17)	25.6	67.7	0.48 [0.31-0.64]	0.0	1.00	1.00 [1.00-1.00]	31.1	60.8	0.31 [0.16-0.47]∗	25.7	66.1	0.45 [0.23-0.68]	38.2	53.7	0.12 [0.01-0.23]
"Immediate hemostatic transfusion" item															
Overall (n = 101)	9.9	84.0	0.80 [0.72-0.89]	0.0	1.00	1.00 [1.00-1.00]	12.0	80.5	0.75 [0.66-0.85]∗	5.2	90.5	0.89 [0.83-0.96]	21.1	67.3	0.51 [0.44-0.58]†
Surgeons (n = 47)	7.3	87.7	0.86 [0.78-0.94]	0.0	1.00	1.00 [1.00-1.00]	8.8	85.0	0.82 [0.73-0.91]∗	3.7	93.2	0.93 [0.86-1.00]	15.6	74.2	0.65 [0.52-0.78]†
Anesthetists (n = 54)	12.2	81.0	0.76 [0.66-0.86]	0.0	1.00	1.00 [1.00-1.00]	14.8	77.0	0.69 [0.58-0.80]∗	6.5	88.2	0.87 [0.79-0.94]	25.9	62.0	0.38 [0.28-0.49]†,‡
Experience ≥10 y (n = 45)	10.6	83.0	0.79 [0.69-0.88]	0.0	1.00	1.00 [1.00-1.00]	12.9	79.3	0.73 [0.63-0.84]∗	6.4	89.5	0.88 [0.80-0.96]	21.5	65.8	0.47 [0.35-0.60]†
Experience <10 y (n = 56)	8.9	85.7	0.83 [0.74-0.92]	0.0	1.00	1.00 [1.00-1.00]	10.8	82.6	0.79 [0.69-0.88]∗	4.4	92.8	0.92 [0.86-0.99]	19.3	69.0	0.55 [0.44-0.66]†
Activity <100/y (n = 29)	14.2	79.9	0.73 [0.61-0.86]	0.0	1.00	1.00 [1.00-1.00]	17.2	75.5	0.66 [0.52-0.80]∗	5.2	90.2	0.89 [0.82-0.96]	33.3	55.9	0.21 [0.10-0.31]†,‡
Activity 100-200/y (n = 37)	8.7	84.8	0.82 [0.75-0.89]	0.0	1.00	1.00 [1.00-1.00]	10.6	81.6	0.77 [0.69-0.85]∗	6.1	88.8	0.87 [0.80-0.95]	16.7	71.9	0.61 [0.53-0.69]†
Activity > 200/y (n = 35)	7.6	87.4	0.85 [0.77-0.94]	0.0	1.00	1.00 [1.00-1.00]	9.2	84.7	0.82 [0.72-0.91]∗	4.3	92.4	0.92 [0.83-1.00]	15.7	0.74	0.65 [0.49-0.81]†
Public center (n = 84)	9.9	83.9	0.80 [0.72-0.89]	0.0	1.00	1.00 [1.00-1.00]	12.1	80.5	0.75 [0.65-0.85]∗	5.1	90.7	0.90 [0.83-0.96]	21.4	66.9	0.50 [0.39-0.61]†
Private center (n = 17)	9.7	83.7	0.80 [0.71-0.89]	0.0	1.00	1.00 [1.00-1.00]	11.8	80.1	0.75 [0.65-0.85]∗	5.9	89.0	0.88 [0.79-0.96]	19.6	68.4	0.54 [0.39-0.69]†

The panel of postcardiopulmonary bypass situations according to visualized bleeding was divided in 3 “normal” (B0) ones and 14 “abnormal” (B+) ones, which included 8 “ambiguously abnormal” (B?) ones and 6 “clearly abnormal” (B!) ones. Here, we detailed the prevalence (f) the observed agreement (Po) and the Gwet's AC1 test (γ) [95% CI].

∗ Significant difference with B0 (for B+).

† Significant difference with B? (for B!).

‡ Significantly lower with other subgroups among each characteristics.

Compared with B0 situations, for which interrater reliability was “excellent” (γ > 0.95 [0.90-1.00]) for each item with a prevalence under 5%, B+ situations showed significantly lower interrater reliability for “abnormal bleeding” and “residual coagulopathy” items, which were “moderate” (γ = 0.43 [0.28-0.58]) and “poor” (γ = 0.27 [0.16-0.37]), respectively. It remained “good” for the item “immediate hemostatic transfusion.”

Among B+ situations, interrater reliability for the “abnormal bleeding” item was “poor” (γ = 0.20 [0.03-0.37]) for B? situations and significantly lower than for B! situations (γ = 0.74 [0.67-0.81]). For the “residual coagulopathy” item, the interrater reliability was “poor” whatever the situation (γ = 0.34 [0.19-0.49] and γ = 0.17 [0.11-0.23], respectively). We noted the respondents with more than 10 years of experience showed a lower inter-reliability facing B! situations (γ = 0.12 [0.05-0.18] vs 0.45 [0.26-0.63] facing B? situations), with a comparable trend among respondents from private centers (γ = 0.12 [0.01-0.23] vs 0.45 [0.23-0.68], respectively). Although the interrater reliability for the item “immediate hemostatic transfusion” remained “excellent” (γ = 0.89 [0.13-0.96]) for B? situations, with an overall prevalence of 5.2%, it was significantly lower for B! situations, for which it was “moderate” (γ = 0.51 [0.44-0.58]), with a prevalence of 21.1%.

### Intra-dyad Agreement

**Figure 2:**
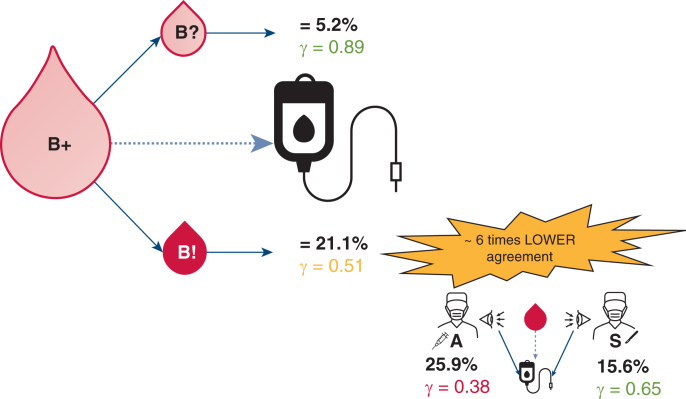
Intra-dyad agreement on immediate hemostatic transfusion in the face of abnormal bleeding situations. Among the abnormal (B+) situations, although ambiguously abnormal (B?) ones showed low “immediate hemostatic transfusion” prevalence with “excellent” interrater reliability (γ > 0.80), clearly abnormal (B!) ones showed greater prevalence with insufficient interrater reliability (γ < 0.60). Agreement between anesthetists (A) was poor (γ < 0.40) and significantly lower than between surgeons (S) with greater prevalence. Intra-dyad agreement was about 6 times lower for clearly abnormal (B!) situations than for ambiguously abnormal (B?) situations.

By comparing interrater reliability by expertise separately, we found there was a slight decrease between anesthetists compared with surgeons for the item “immediate hemostatic transfusion” on overall procedures, which was significant only for B! situations (γ = 0.38 [0.28-0.49] vs γ = 0.65 [0.52-0.78]), with approximately a 1.5-fold more prevalence. We also observed a lower interrater reliability among the respondents who reported performing fewer than 100 major cardiac procedures by year (γ = 0.21 [0.10-0.31] vs γ = 0.61 [0.53-0.69]) compared with respondents who reported performing 100 to 200 major cardiac procedures by year, and γ = 0.65 [0.49-0.81] for respondents with more than 200 major cardiac procedures by year, respectively) (Figure 2).

Compared with B0 situations, B+ situations showed a 13-fold (odds ratio [OR], 13.2 [11.7-14.9]) and 23-fold (OR, 22.7 [19.9-25.9]) lower likelihood of agreement between the 2 physicians on the items “abnormal bleeding” and “residual coagulopathy,” respectively. Compared with B? situations, B! situations showed a 3-fold greater likelihood of agreement between the 2 physicians on the “abnormal bleeding” item (OR, 2.72 [2.58-2.85]). The likelihood of agreement on the “residual coagulopathy” item was slightly lower (OR, 0.79 [0.76-0.83]), whereas it was 6-fold lower (OR, 0.18 [0.17-0.19]) for the item “immediate hemostatic transfusion.”

## Discussion

In clinical practice, interrater reliability should be “good” for subjective diagnoses, such as abnormal bleeding or residual coagulopathy, and “excellent” for objective or critical decisions like hemostatic transfusion. In this nationwide study, overall reliability indicated that clinical practice remained suboptimal regarding individual point of view (Figure 1). Although our method for selecting and defining post-CPB bleeding situations was subjective and potentially biased (Appendix E1), the interrater reliability observed for each category—particularly the lowest consistency for the B? situations—subsequently supported our shared ability to subjectively recognize the extreme situations while highlighting our limited ability to define some intermediate ones.

Because residual coagulopathy is difficult to identify at a glance, immediate hemostatic transfusion was inconsistent even in clear bleeding situations, with a risk of low intra-dyad agreement between anesthetists and surgeons. Conversely, interrater reliability for the item “immediate hemostatic transfusion” was greater in the B? situations, likely because respondents adopted a watch-and-wait approach, resulting in a low endorsement rate (∼5%). In contrast, the “chaotic” B! situations appear to be at the greatest risk for human error: they were associated with a high rate but low consistency of immediate hemostatic therapy decisions, and they would benefit from standardized management to prevent both delayed and inappropriate hemostatic transfusion.

The 3 assessed items—perception of abnormal bleeding, understanding of residual coagulopathy, and anticipation of severe bleeding—represent the stepwise levels of situational awareness,^14^ a core nontechnical skill required by both surgeons and anesthetists for effective teamwork in the operating room.^12^^,^^13^ This cognitive process is schematized by decision-making algorithms. Although laboratory and viscoelastic testing improve understanding of coagulopathy, optimal transfusion decisions first require accurate perception, because all the decision-making algorithms remind one to correct abnormal values only in cases of abnormal bleeding. Thus, training clinicians to differentiate normal from abnormal situations appears necessary—and potentially achievable—but it still relies largely on apprenticeship-style learning and is therefore highly dependent on local institutional culture.

In open surgery, bleeding is common and follows fuzzy logic.^16^ Algorithms usually define abnormal bleeding as “excessive,” “diffuse,” or “microvascular.” To reduce subjectivity, surgeons developed closure checklists^17^^,^^18^ and the 5-minute test.^19^ Using a checklist, surgeons assessed all potential sources of “localized” and “macrovascular” bleeding at cardiotomy sites and along the chest wall. The 5-minute test provides information on bleeding rate and localization by weighing surgical gauze sponges, and “excessive” bleeding is defined using a threshold of 60 to 100 g. Regardless of the tool, their use requires dedicated time to manage intraoperative bleeding. Ultimately, rather than relying on a single ideal tool, combining several approaches may be the best way to improve our understanding of intraoperative bleeding.^20^ A visual scale and standardized wound video clips have since been proposed to enhance rapid bleeding assessment,^21^ and this tool was integrated by Karkouti and colleagues^22^ into the FARES-II study algorithms. This tool correlates bleeding flow with the surgical source across different types of vascular injuries, ranging from capillaries to the vena cava. Because it was not specific to post-CPB bleeding, we used real-life sternotomy video clips after antagonization to better reflect daily practice.^23^^,^^24^

Operative bleeding is dynamic and influenced by multiple factors related to surgical technique and hemostatic management. This complexity increases uncertainty, especially as post-CPB bleeding combines coagulopathy and surgical sources. Despite structured decision-making algorithms, actions in real time may not always ensure patient safety. The “prevention is better than cure” principle may justify unnecessary transfusion, deviating from primum non nocere at acceptable risk and cost. Anesthetists from high-transfusion centers report greater satisfaction,^25^ likely reflecting a tendency toward immediate hemostatic transfusion when bleeding appears clearly abnormal. In our study, interrater reliability for deciding on immediate hemostatic transfusion was significantly lower among anesthetists than among surgeons. Although we could not directly compare response rates between anesthesiologists and surgeons, we observed an approximately 10% difference. Beyond years of experience, anesthetists reported a lower individual annual volume of major cardiac procedures, and clinicians performing fewer than 100 procedures per year tended to choose immediate hemostatic transfusion more often, with poorer—and significantly lower—interrater reliability than those with greater individual case volume. Unexpectedly, agreement on the item “residual coagulopathy” in clearly bleeding situations was lower among the most experienced respondents. We hypothesized that local institutional culture may substantially influence clinical judgment, as suggested by a similar trend according to center affiliation. Given that the impact of case mix on transfusion variability is estimated at approximately 10% to 20%,^26^^,^^27^ and assuming comparable technical resources across respondents, organizational and human factors likely remain major drivers of variability in transfusion practice and, by extension, in bleeding management. In addition to interphysician variability,^14^^,^^25^ centers have a major impact, and uniformization could correct misguided practices.^27^, ^28^, ^29^

Decision-making is closely related to the situation the decision-maker is facing.^30^ When confronted with a complex situation, there is no gold standard and a spectrum of practices is possible, ranging from “satisficing” to optimization. A “prevent-to-act” approach, based on a cause-and-effect algorithmic model, leads to liberal transfusion at the slightest perceived risk, with the goal of avoiding severe bleeding—even if that means treating a presumed coagulopathy in the absence of objective evidence. The corollary is greater-than-necessary transfusion, especially because the cause-and-effect relationship is oversimplified, exposing patients to avoidable risk while also increasing health care costs and reducing blood product inventories, which may already be limited. The greater satisfaction reported by anesthetists from the highest-transfusing centers compared with those from lower-transfusing centers reflects this tolerance of added risk and added cost.^25^ To reach efficiency, optimization instead favors a “monitor-and-adapt” approach, using transfusion only when it is actually beneficial. This strategy requires a high level of situational awareness from each team member^14^^,^^31^ and adds substantial cognitive load at a critical moment in the operation.^12^^,^^13^ Surgeons, who proceed step-by-step and have access to a broad range of hemostatic techniques and technologies, should play a leading role in hemostatic transfusion management. Recent algorithms emphasize the need for their active participation in bleeding assessment.^32^^,^^33^

Finally, in an era in which patient blood management is a keystone in patient safety during any invasive procedure,^3^^,^^34^ the inappropriate use of hemostatic blood components should be avoided, as the packed red blood cells use should be restrictive.^6^, ^7^, ^8^ In addition to new hemostatic treatments, surgeons and anesthetists should improve their nontechnical skills by developing not cognitive tools but also specific education and training program. This study showed individual judgment is by itself insufficient and, when faced with abnormal bleeding, a shared mental model between the surgeon and the anesthetist could reduce inappropriate use of hemostatic blood components by compensating for the clinical information each may lack individually. By sharing their analyses, each specialist enhances his/her and the other's situational awareness, whereas poor communication may lead to confusion—or even conflict—to the detriment of patient safety. Decision-making algorithms could be used not only to guide hemostatic transfusion but also to script behavior and communication between them.

### Strengths and Limitations

To our knowledge, this is the first study to assess interrater reliability in post-CPB bleeding evaluation using real intraoperative video clips, emphasizing the role of human factors and situational awareness. Brief clips simulated quick intraoperative judgment under time constraints, consistent with previous models. Standardized clinical scenarios and advanced simulation could better assess how bleeding affects transfusion decisions and support targeted physician training.

A survey capturing more than 10% of the target population may be considered representative. Unlike for surgeons, we were not able to distinguish anesthesiologists involved in cardiac surgery from those whose practice is exclusively thoracic or vascular; therefore, the response rate for the anesthesiology group was mathematically underestimated. Nevertheless, the distribution of center affiliation matched the national distribution of cardiac surgery centers in France, with approximately 87% (55/63) being public centers.

This study is limited by the lack of real-world postoperative outcomes, such as morbidity/mortality or transfusion requirements, which are multifactorial and not driven by bleeding alone. Because no gold standard exists, we did not aim to determine who was right or wrong; instead, we measured interrater reliability rather than comparing response rates. We assumed that high consistency reflects acquired skills, whereas low consistency suggests a skills gap—regrettable in a situation encountered almost daily. Although multiple approaches may be appropriate, we argue that standardization could reduce potentially risky behavior. Finally, we did not collect justifications for respondents’ answers; future work exploring the cognitive frameworks used when facing abnormal bleeding may help us develop decision-support tools and training programs to better address this issue.

## Conclusions

Abnormal bleeding in cardiac surgery remains a significant clinical challenge. Variability in its assessment can impede optimal hemostatic transfusion management. Enhancing situational awareness through structured cognitive tools and targeted training may strengthen patient blood-management strategies. Promoting interdisciplinary teamwork and a shared mental model between surgeons and anesthetists fosters more consistent decision-making.

### Declaration of Generative AI and AI-Assisted Technologies in the Writing Process

During the preparation of this work, the authors used ChatGPT-5 to assist with translation and language refinement. After using this tool/service, the authors reviewed and edited the content as needed and take full responsibility for the content of the publication.

## Conflict of Interest Statement

The authors reported no conflicts of interest.

The *Journal* policy requires editors and reviewers to disclose conflicts of interest and to decline handling or reviewing manuscripts for which they may have a conflict of interest. The editors and reviewers of this article have no conflicts of interest.

## Figures and Tables

**Figure:**
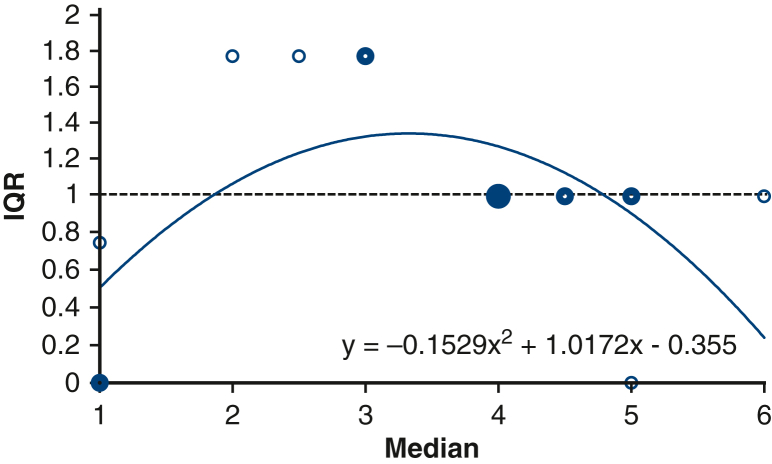


## Appendix E1 Video Clips and Definition of Bleeding Situations

### Video Clip Database

Over a 1-month period, we recorded videos of the operative field from consecutive open cardiac surgery cases using a Google Pixel 2 smartphone (Google LLC), positioned from the anesthesiologist's perspective with a 3.0× zoom.

All recordings were made between 10 and 20 minutes after weaning from cardiopulmonary bypass (CPB), after the planned hemostatic therapy had been administered, including heparin reversal and any previously ordered products. In all cases, temperature (>36 °C), pH (>7.2), and ionized calcium (>1.0 mmol/L) were optimized, and antifibrinolytic therapy with tranexamic acid was continuously infused.

Because recordings were obtained under real-life conditions, each video was segmented into 10- to 20-second clips in which the operative field was clearly visible and not obscured by the operators’ hand movements. All audio was removed. Video editing was performed using Adobe Premiere Rush (Adobe).

### Selection of the Panel for the Questionnaire

One author (A.S.) selected a set of at least 10 sequences representing a spectrum of subjectively different bleeding patterns. Assuming that the survey should last no more than 5 minutes in order to achieve an adequate response rate, the final panel included 17 video clips. This selection was validated by a dyad of experts from the same center—one surgeon (O.C.) and one anesthesiologist (P.A.)—each with more than 20 years of experience in cardiac surgery.

### Semiarbitrary Predefining of the Bleeding Situations

First, the 2 experts independently judged whether the bleeding was “normal” or “abnormal” regardless of the presumed cause or the therapy they would suggest. This resulted in 3 categories of agreement regarding bleeding perception: “clearly normal” (normal/normal agreement between the two experts), “ambiguously abnormal” (normal/abnormal disagreement), and “clearly abnormal” (abnormal/abnormal agreement).

Subsequently, anesthesiologists (n = 8) and surgeons (n = 6) from the same center, with varying levels of expertise—from residents to senior clinicians—judged the normality/abnormality of the bleeding, regardless of the presumed cause or the therapy they would suggest, using a 6-point balanced Likert scale with a unitary index, as presented to follow:

1: clearly normal
2: rather normal
3: maybe normal
4: maybe abnormal
5: rather abnormal
6: clearly abnormal

Because no previously validated scale existed, this scale was developed according to the following principles: (1) although a scale may include anywhere from 3 to 100 (or even more) categories, an optimal scale should include no more than 11 levels (0-10) and ideally be between 5 and 7 points; and (2) an even number of points forces respondents to make a choice, whereas an odd number risks overuse of a neutral response. Kendall's W coefficient was 0.77, indicating good inter-rater reliability, and was considered sufficient for validation given a qualitative judgment threshold of 0.60.

Using this index, score dispersion was displayed as the interquartile range (IQR) around the median score for each video on a graph. Circle thickness was proportional to the number of video clips sharing the same characteristics.

We Plotted a third-degree polynomial regression curve (solid line). this curve crossed the line at IQR = 1 (dotted line) at abscissa values of 1.8 and 4.8

Video clips were defined as follows: (Vimeo link is available in addition):

The 3 video clips with a median less 1.8 presented an IQR strictly inferior to 1 and were considered as “clearly normal” (B0) situations:

#01 (median = 1.0/IQR = 0.0) https://vimeo.com/940019522.

#10 (median = 1.0/IQR = 0.0) https://vimeo.com/988494754.

#17 (median = 1.0/IQR = 0.75) https://vimeo.com/988471989.

The 8 video clips with median between 1.8 and 4.8 presented an IQR superior or equal to 1 and were considered as “ambiguously abnormal” (B?)

#03 (median 4.0/IQR 1.0) https://vimeo.com/940135925.

#05 (median 4.0/IQR 1.0) https://vimeo.com/940143900.

#06 (median 4.0/IQR 1.0) https://vimeo.com/940149137.

#11 (median 2.5/IQR 1.75) https://vimeo.com/988491397.

#12 (median 2.0/IQR 1.75) https://vimeo.com/988446988.

#13 (median 3.0/IQR 1.75) https://vimeo.com/988456934.

#15 (median 4.0/IQR 1.0) https://vimeo.com/988465862.

#16 (median 3.0/IQR 1.75) https://vimeo.com/988468028.

The 6 video clips with median over 4.8 presented an IQR inferior or equal to 1 and were considered as “clearly abnormal” (B!)

#02 (median 6.0/IQR 1.0) https://vimeo.com/940127096.

#04 (median 5.0/IQR 1.0) https://vimeo.com/940141667.

#07 (median 5.0/IQR 0.0) https://vimeo.com/940154259.

#08 (median 5.0/IQR 1.0) https://vimeo.com/940162400.

#09 (median 4.5/IQR 1.0) https://vimeo.com/987953487.

#14 (median 4.5/IQR 1.0) https://vimeo.com/988460618.

## Supplementary Data

Video 1 Intraoperative view #1. Video available at: https://www.jtcvs.org/article/S2666-2736(26)00139-7/fulltext.

Video 2 Intraoperative view #2. Video available at: https://www.jtcvs.org/article/S2666-2736(26)00139-7/fulltext.

Video 3 Intraoperative view #3. Video available at: https://www.jtcvs.org/article/S2666-2736(26)00139-7/fulltext.

Video 4 Intraoperative view #4. Video available at: https://www.jtcvs.org/article/S2666-2736(26)00139-7/fulltext.

Video 5 Intraoperative view #5. Video available at: https://www.jtcvs.org/article/S2666-2736(26)00139-7/fulltext.

Video 6 Intraoperative view #6. Video available at: https://www.jtcvs.org/article/PII/fulltext.

Video 7 Intraoperative view #7. Video available at: https://www.jtcvs.org/article/S2666-2736(26)00139-7/fulltext.

Video 8 Intraoperative view #8. Video available at: https://www.jtcvs.org/article/S2666-2736(26)00139-7/fulltext.

Video 9 Intraoperative view #9. Video available at: https://www.jtcvs.org/article/S2666-2736(26)00139-7/fulltext.

Video 10 Intraoperative view #10. Video available at: https://www.jtcvs.org/article/S2666-2736(26)00139-7/fulltext.

Video 11 Intraoperative view #11. Video available at: https://www.jtcvs.org/article/S2666-2736(26)00139-7/fulltext.

Video 12 Intraoperative view #12. Video available at: https://www.jtcvs.org/article/S2666-2736(26)00139-7/fulltext.

Video 13 Intraoperative view #13. Video available at: https://www.jtcvs.org/article/S2666-2736(26)00139-7/fulltext.

Video 14 Intraoperative view #14. Video available at: https://www.jtcvs.org/article/S2666-2736(26)00139-7/fulltext.

Video 15 Intraoperative view #15. Video available at: https://www.jtcvs.org/article/S2666-2736(26)00139-7/fulltext.

Video 16 Intraoperative view #16. Video available at: https://www.jtcvs.org/article/S2666-2736(26)00139-7/fulltext.

Video 17 Intraoperative view #17. Video available at: https://www.jtcvs.org/article/S2666-2736(26)00139-7/fulltext.

## Appendix E2 Nationwide Questionnaire (English Version)

Notes (1) for readers of the *Journal*:

Because the questionnaire was addressed to French cardiac surgeons and anesthetists, the original text was written in French. Here, an English translation of the questionnaire is provided below for readers of the *Journal*. The introductory section was intentionally omitted from this Appendix E1. The questionnaire was divided into 2 parts.

### Part 1: Respondent Characteristics

(only one answer per question)

1. What is your specialty:
  ☐ Surgeon.
  ☐ Anesthetist
2. What is your length of expertise
  ☐ <10 years (included resident and fellow)
  ☐ ≥10 years
3. How many major cardiac procedures (including off-pump coronary artery bypass grafting) do you perform in 2024?
  ☐ <100 procedures
  ☐ 100-200 procedures
  ☐ >200 procedures
4. What is the affiliation of your center?
  ☐ Public
  ☐ Private

### Part 2: Review of video clips.

∖!∖ Please read before starting∖!∖

All sequences were recorded **AFTER cardiopulmonary bypass (CPB), between 10 and 20 minutes AFTER reversal** (sometimes with additional hemostatic products that had been planned preoperatively or administered during CPB). Thus, both surgical and medical hemostasis are assumed to have been corrected.

The videos are presented **WITHOUT clinical context** (you may only be able to infer it from procedural clues, such as aortic surgery, coronary artery bypass grafting, etc.). Temperature (>36.0 °C), pH (>7.2), and ionized calcium (>1.0 mmol/L) are considered optimized and antifibrinolytic therapy is continuously perfused.

You are asked to watch **THE ENTIRE video (∼10–20 seconds)** and judge the bleeding as **“NORMAL”** or **“ABNORMAL”** (only one response)

∖!∖ Each video can be viewed only once ∖!∖

➢ If you select “NORMAL,” you authorize chest closure and consider that the bleeding is controlled **WITHOUT ANY additional intervention**, either surgical or anesthetic, and that it will not result in clinically significant blood loss.

You will then proceed to the next video clip.

➢ If you select “ABNORMAL,” a second page appears and you are asked whether:
  - you suppose that **RESIDUAL COAGULOPATHY** persists, regardless of the hemostatic therapy administered at CPB weaning, the biological disorder, and the possible presence of a concomitant surgical source.
  - you used **IMMEDIATE HEMOSTATIC THERAPY** with labile or plasma-derived hemostatic blood components—such as platelet concentrates, fresh frozen plasma, prothrombin complex concentrate, fibrinogen concentrate, etc—**BEFORE waiting for additional information**, such as laboratory test results or colleagues' opinions.

You will then proceed to the next video clip.

Notes (2) for readers of the *Journal*:

The video clips (N = 17) were displayed in a randomized order for each respondent, following the same display pattern as presented above. The video clips are available on the Vimeo platform via a restricted-access link. The corresponding links for each video clip, according to predefined bleeding situations (cf. Appendix E1), are provided. Each video clip was assigned an identification number.

Clearly normal “B0” (N = 3) Ambiguously abnormal “B?” (N = 8)∗

#01 https://vimeo.com/940019522 #03 https://vimeo.com/940135925.

#10 https://vimeo.com/988494754 #05 https://vimeo.com/940143900.

#17 https://vimeo.com/988471989 #06 https://vimeo.com/940149137.

#11 https://vimeo.com/988491397.

Clearly abnormal “B!” (N = 6)∗ #12 https://vimeo.com/988446988.

#02 https://vimeo.com/940127096 #13 https://vimeo.com/988456934.

#04 https://vimeo.com/940141667 #15 https://vimeo.com/988465862.

#07 https://vimeo.com/940154259 #16 https://vimeo.com/988468028.

#08 https://vimeo.com/940162400.

#09 https://vimeo.com/987953487.

#14 https://vimeo.com/988460618.
