# Supplementary figures and images for: Abnormal bleeding in the cardiac operating room: An observational study of interrater reliability between anesthetists and surgeons
Source: JTCVS Open. 2026 Mar 6;31:101716. doi: 10.1016/j.xjon.2026.101716 (PMC13316329; doi:10.1016/j.xjon.2026.101716)

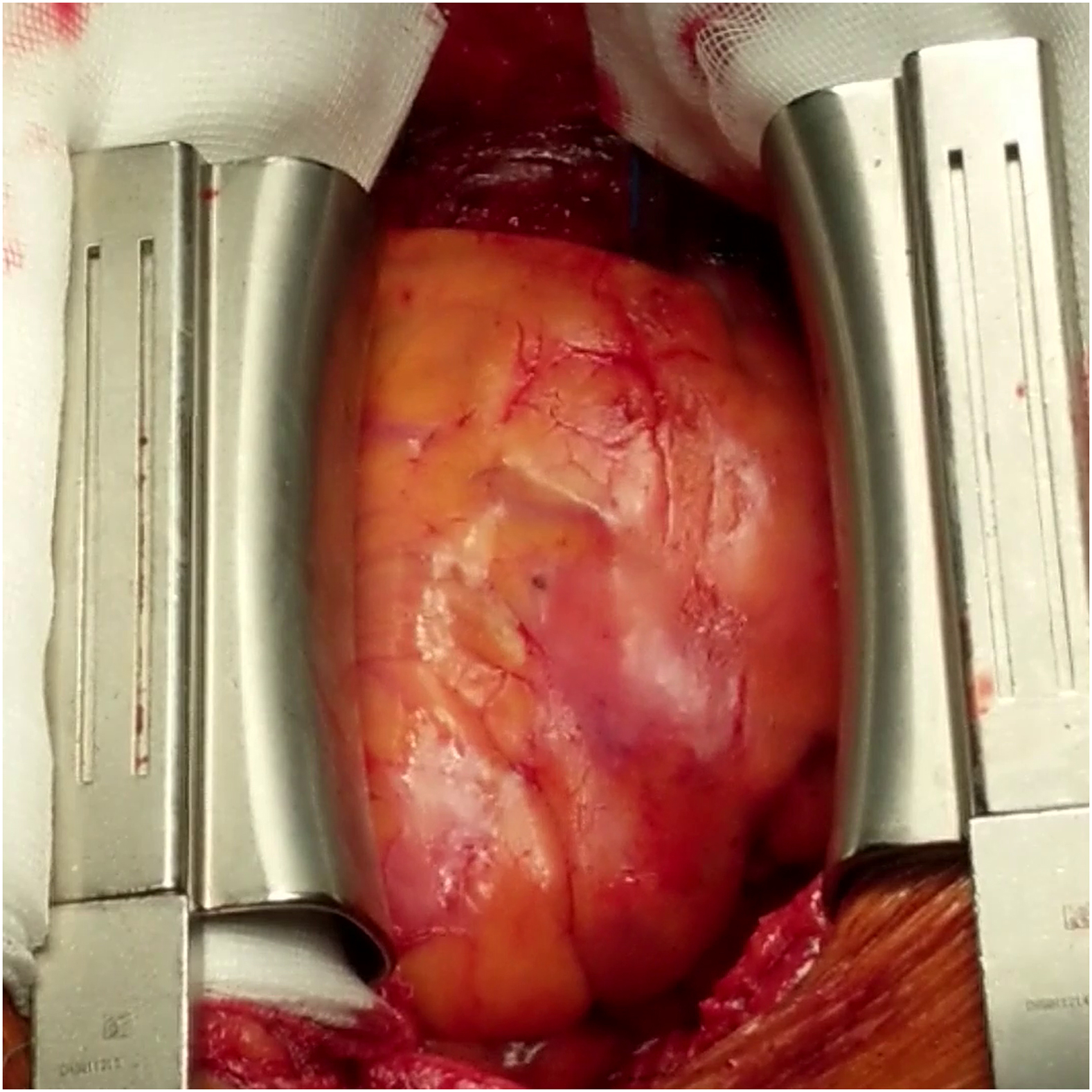

Supplement: Video 1 — Intraoperative view #1. Video available at: https://www.jtcvs.org/article/S2666-2736(26)00139-7/fulltext. [file fx2.jpg]

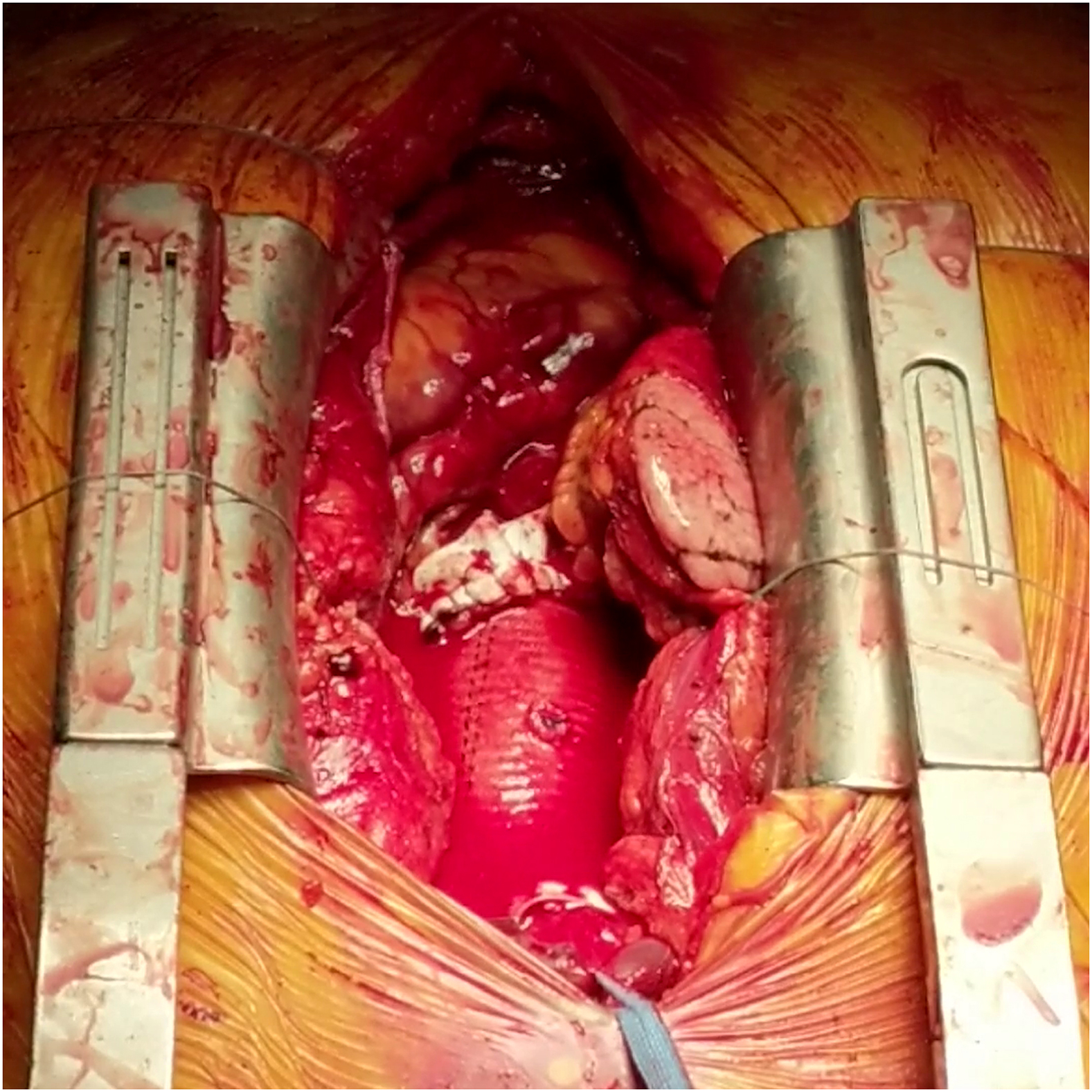

Supplement: Video 2 — Intraoperative view #2. Video available at: https://www.jtcvs.org/article/S2666-2736(26)00139-7/fulltext. [file fx3.jpg]

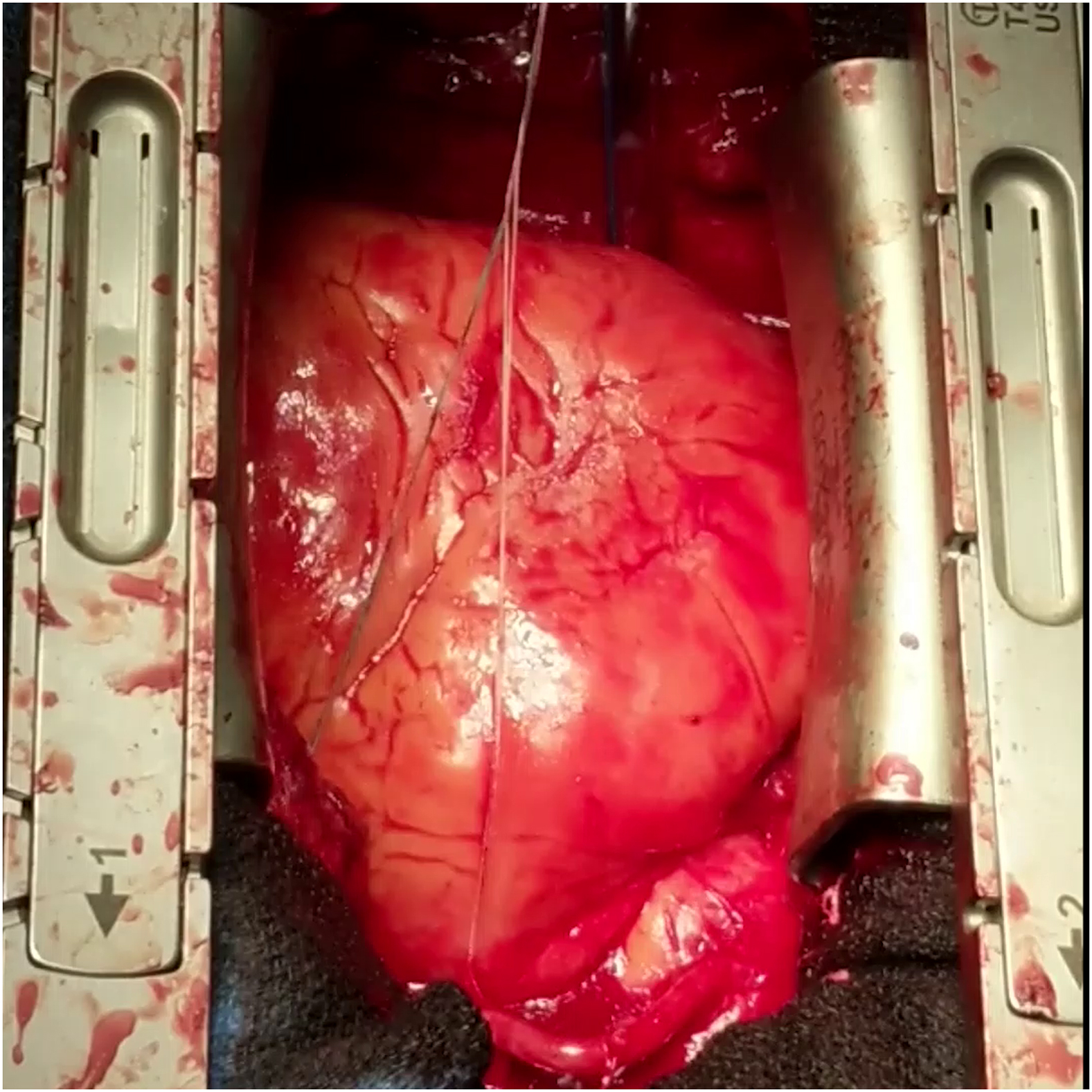

Supplement: Video 3 — Intraoperative view #3. Video available at: https://www.jtcvs.org/article/S2666-2736(26)00139-7/fulltext. [file fx4.jpg]

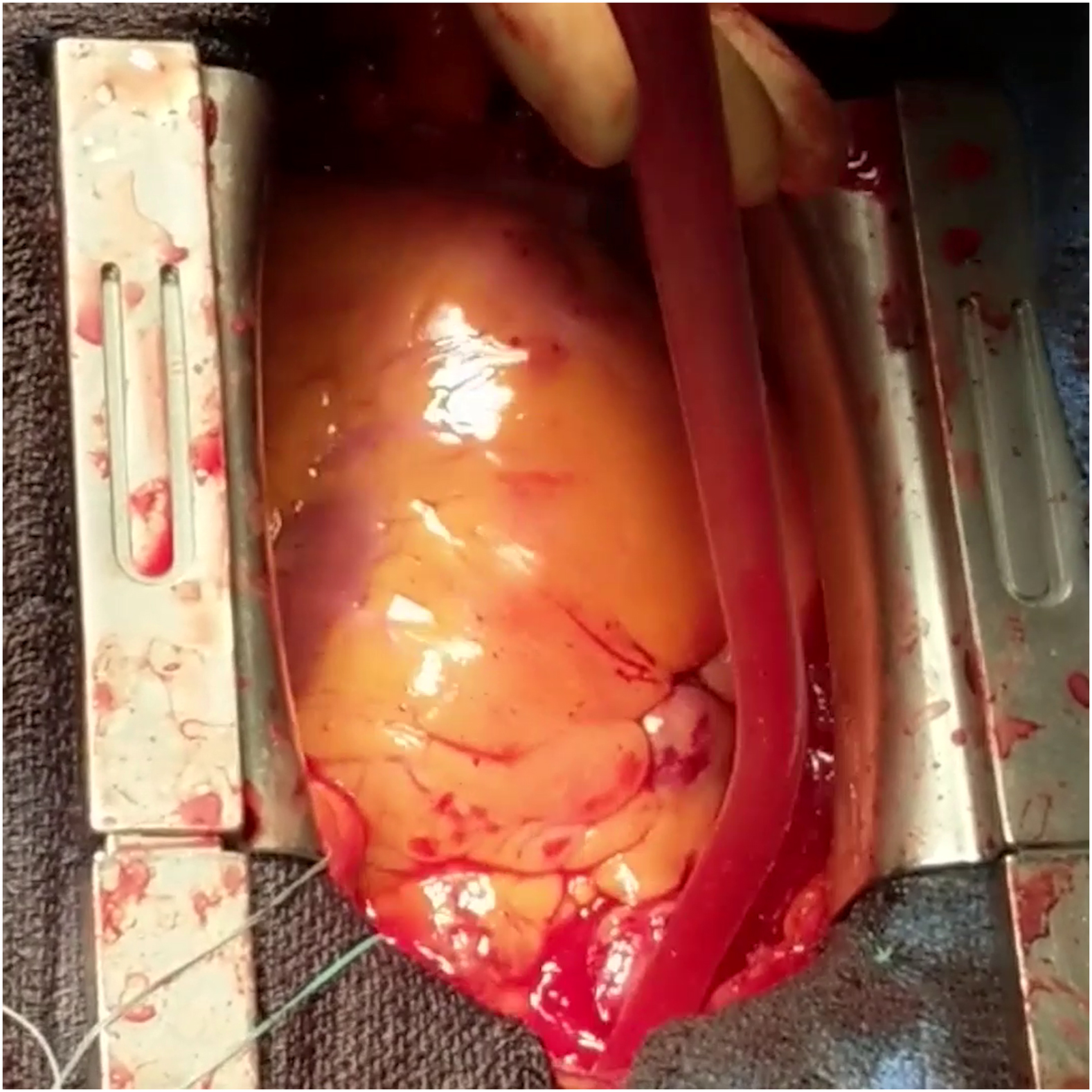

Supplement: Video 4 — Intraoperative view #4. Video available at: https://www.jtcvs.org/article/S2666-2736(26)00139-7/fulltext. [file fx5.jpg]

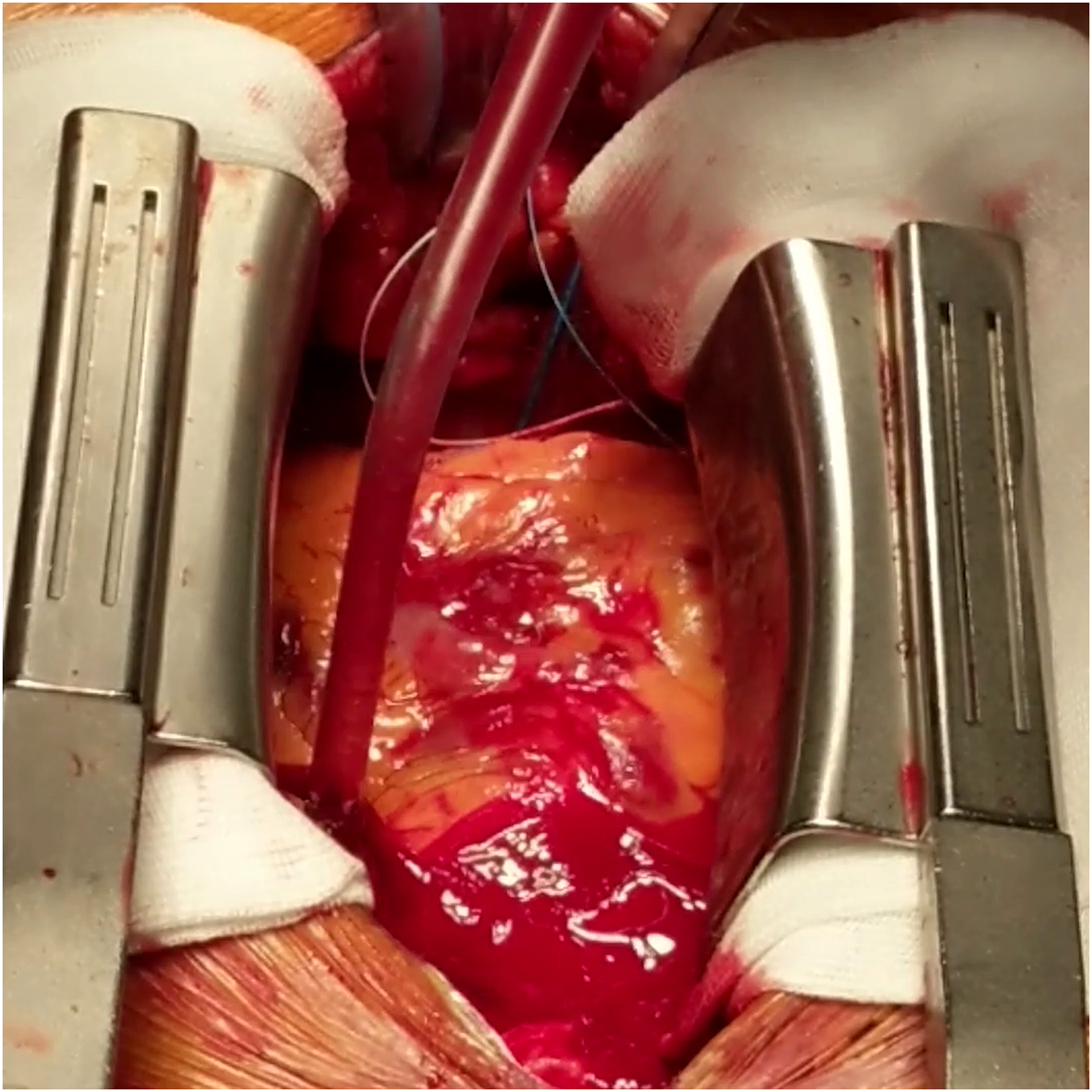

Supplement: Video 5 — Intraoperative view #5. Video available at: https://www.jtcvs.org/article/S2666-2736(26)00139-7/fulltext. [file fx6.jpg]

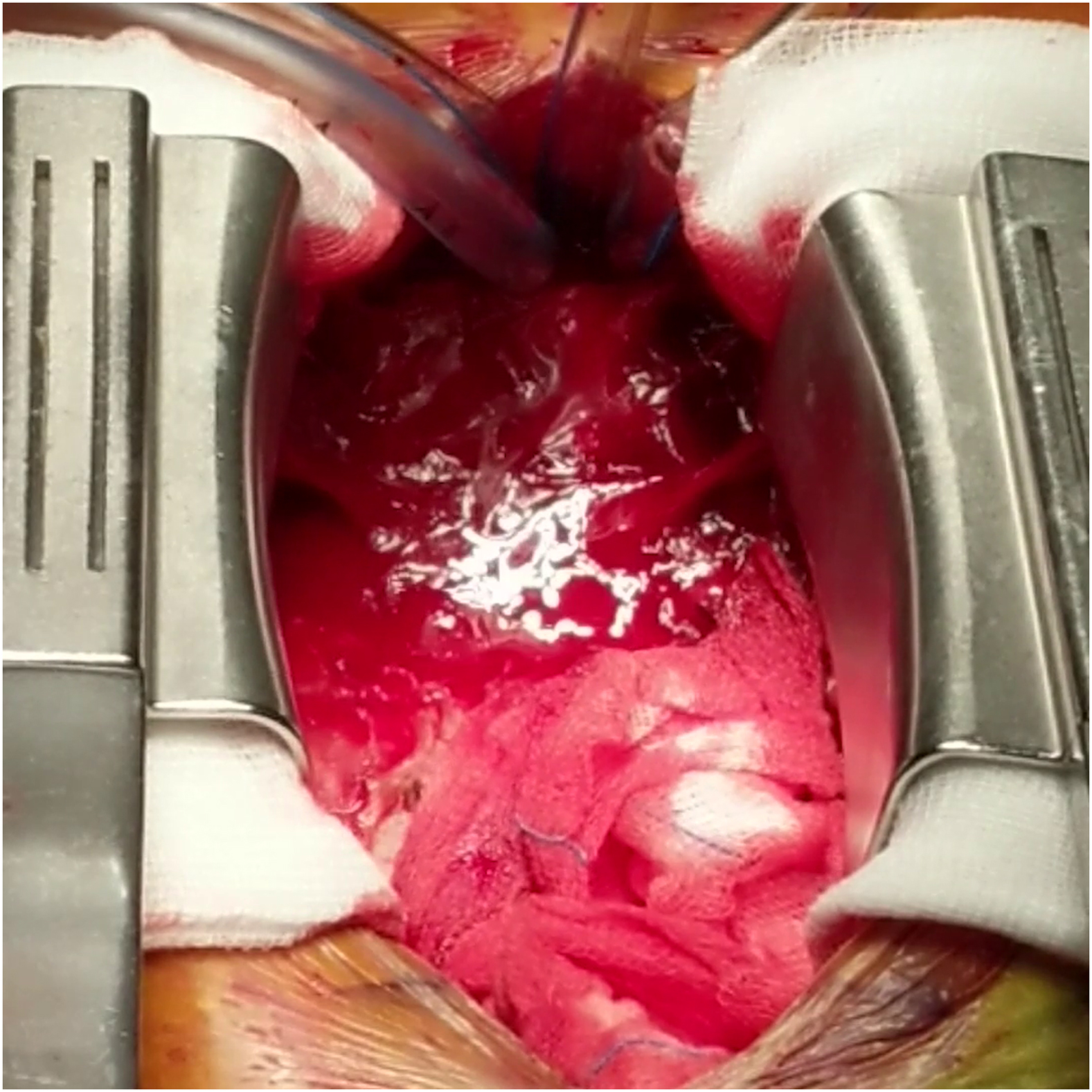

Supplement: Video 6 — Intraoperative view #6. Video available at: https://www.jtcvs.org/article/PII/fulltext. [file fx7.jpg]

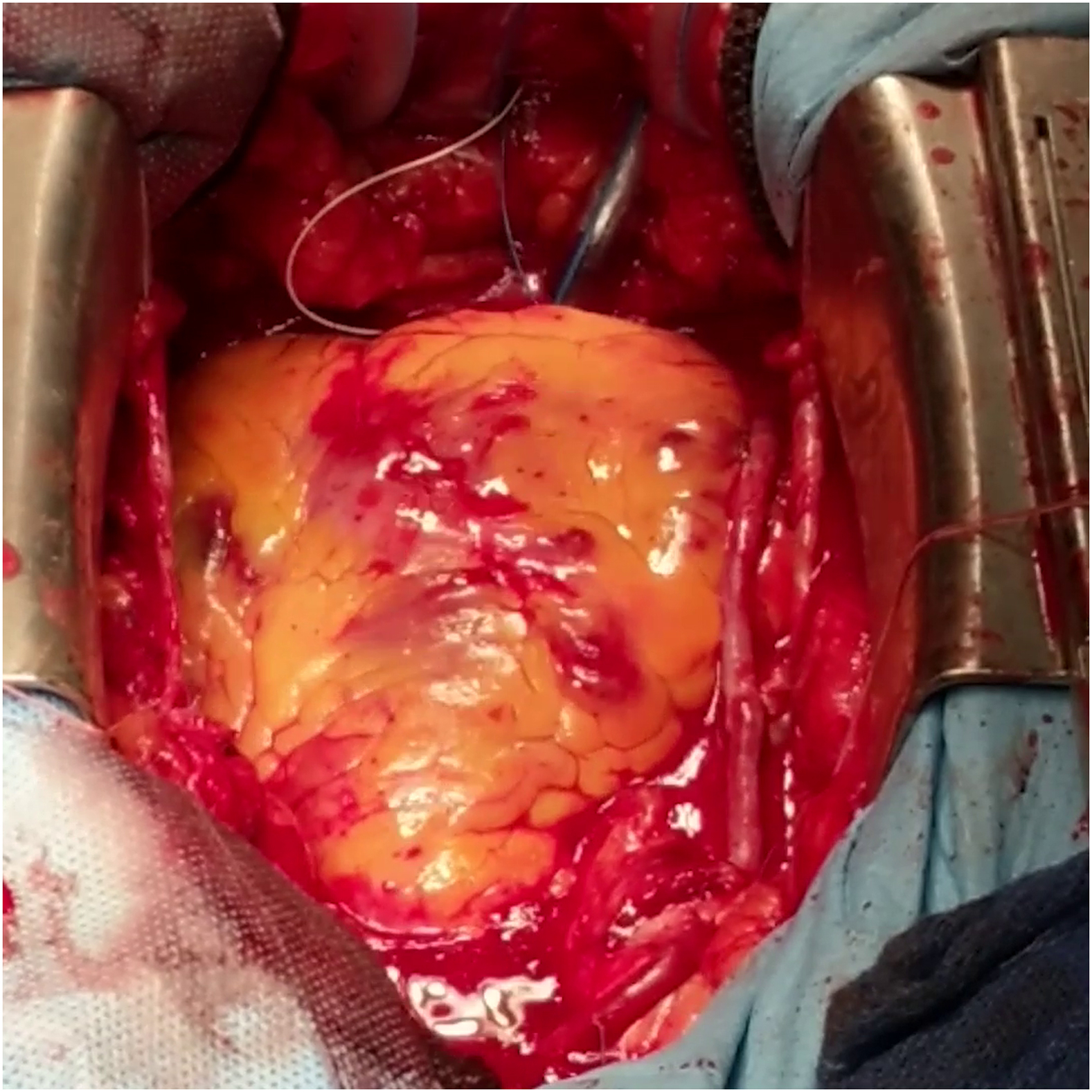

Supplement: Video 7 — Intraoperative view #7. Video available at: https://www.jtcvs.org/article/S2666-2736(26)00139-7/fulltext. [file fx8.jpg]

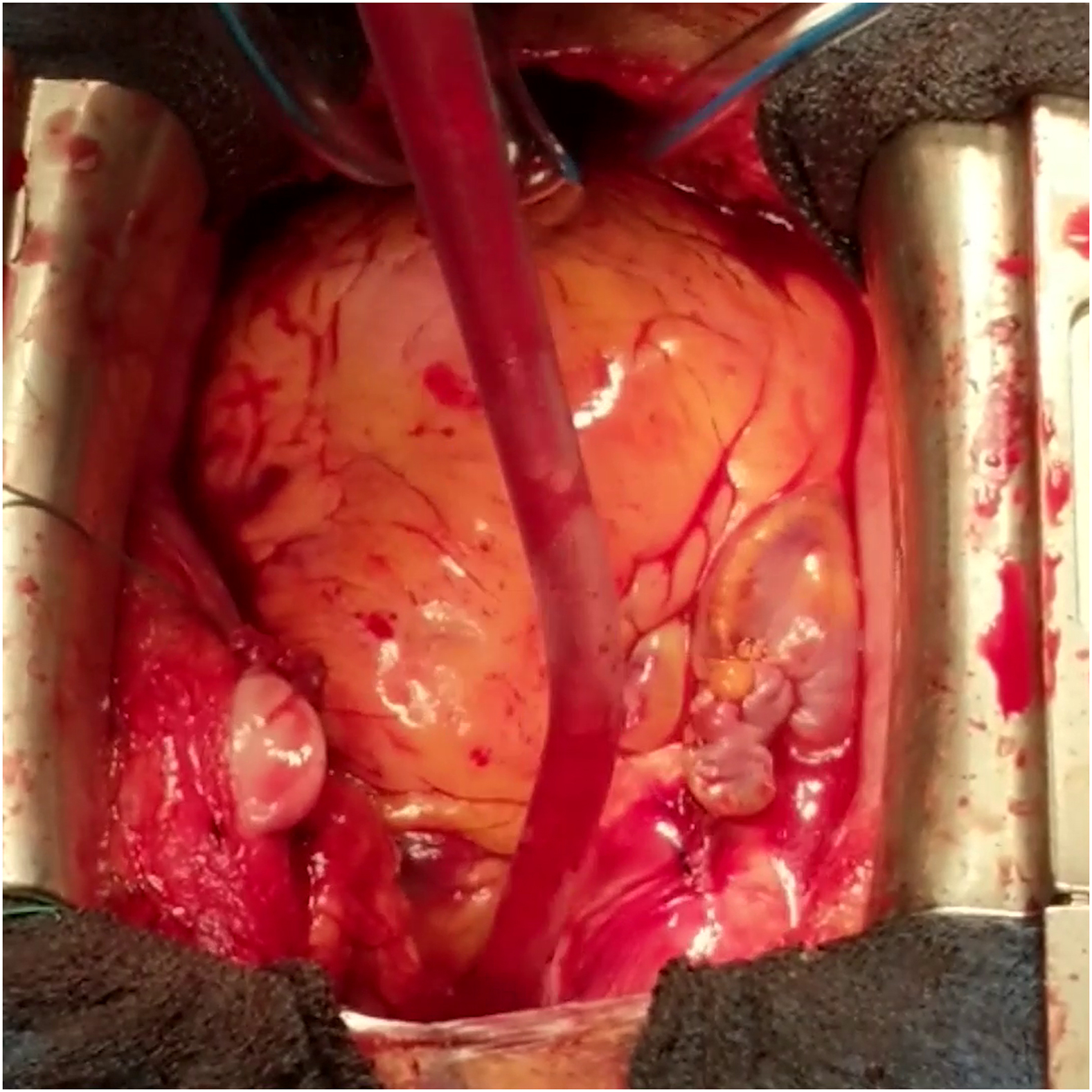

Supplement: Video 8 — Intraoperative view #8. Video available at: https://www.jtcvs.org/article/S2666-2736(26)00139-7/fulltext. [file fx9.jpg]

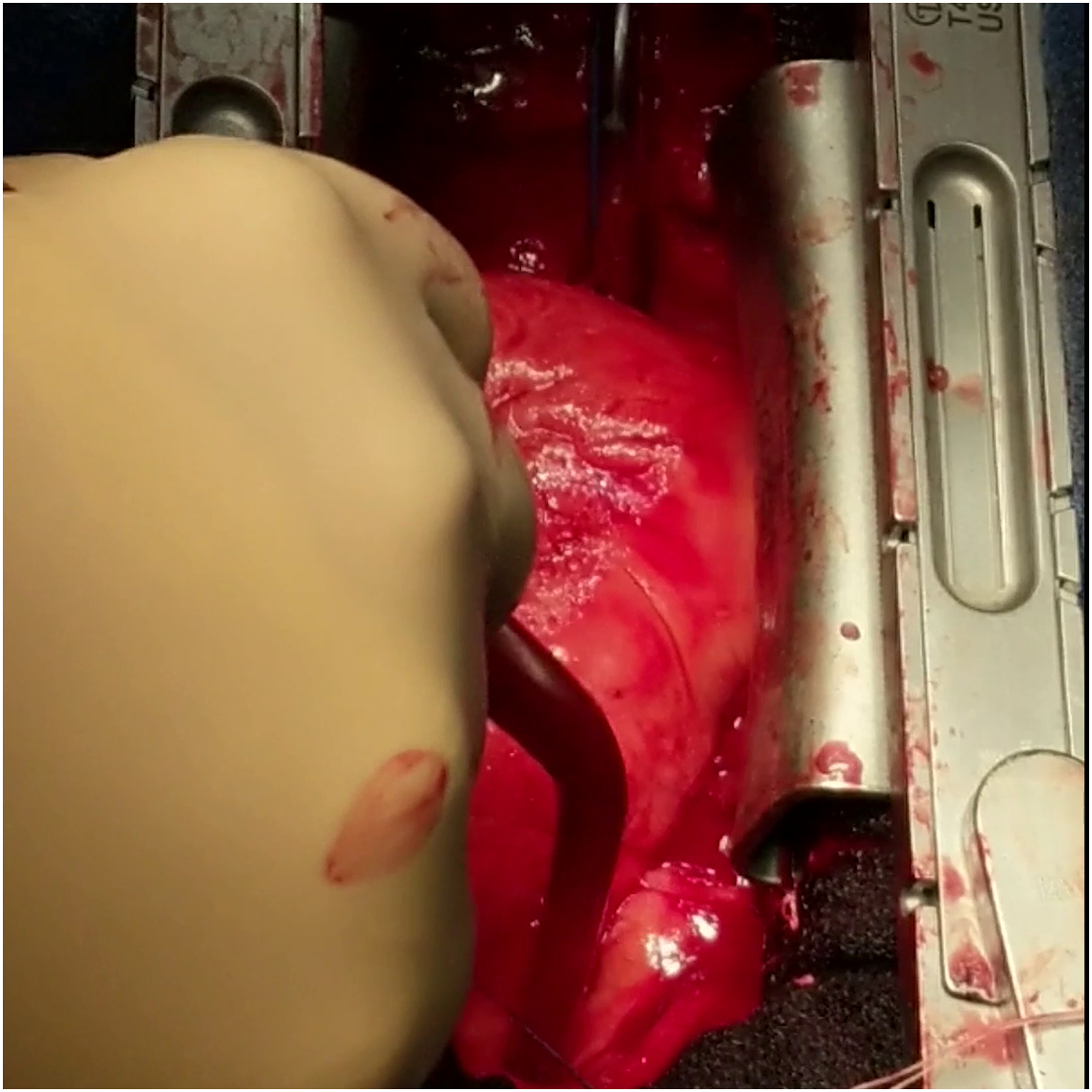

Supplement: Video 9 — Intraoperative view #9. Video available at: https://www.jtcvs.org/article/S2666-2736(26)00139-7/fulltext. [file fx10.jpg]

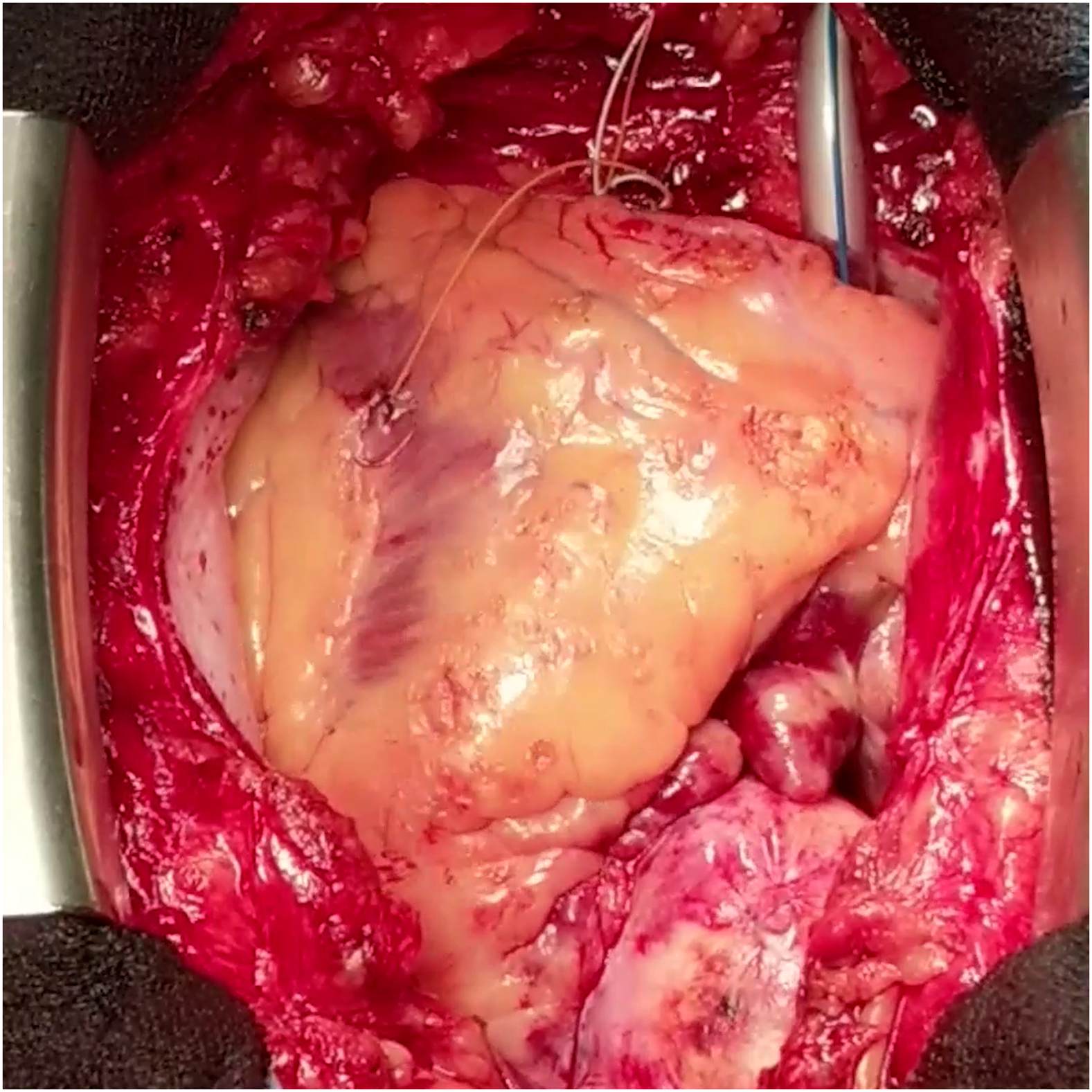

Supplement: Video 10 — Intraoperative view #10. Video available at: https://www.jtcvs.org/article/S2666-2736(26)00139-7/fulltext. [file fx11.jpg]

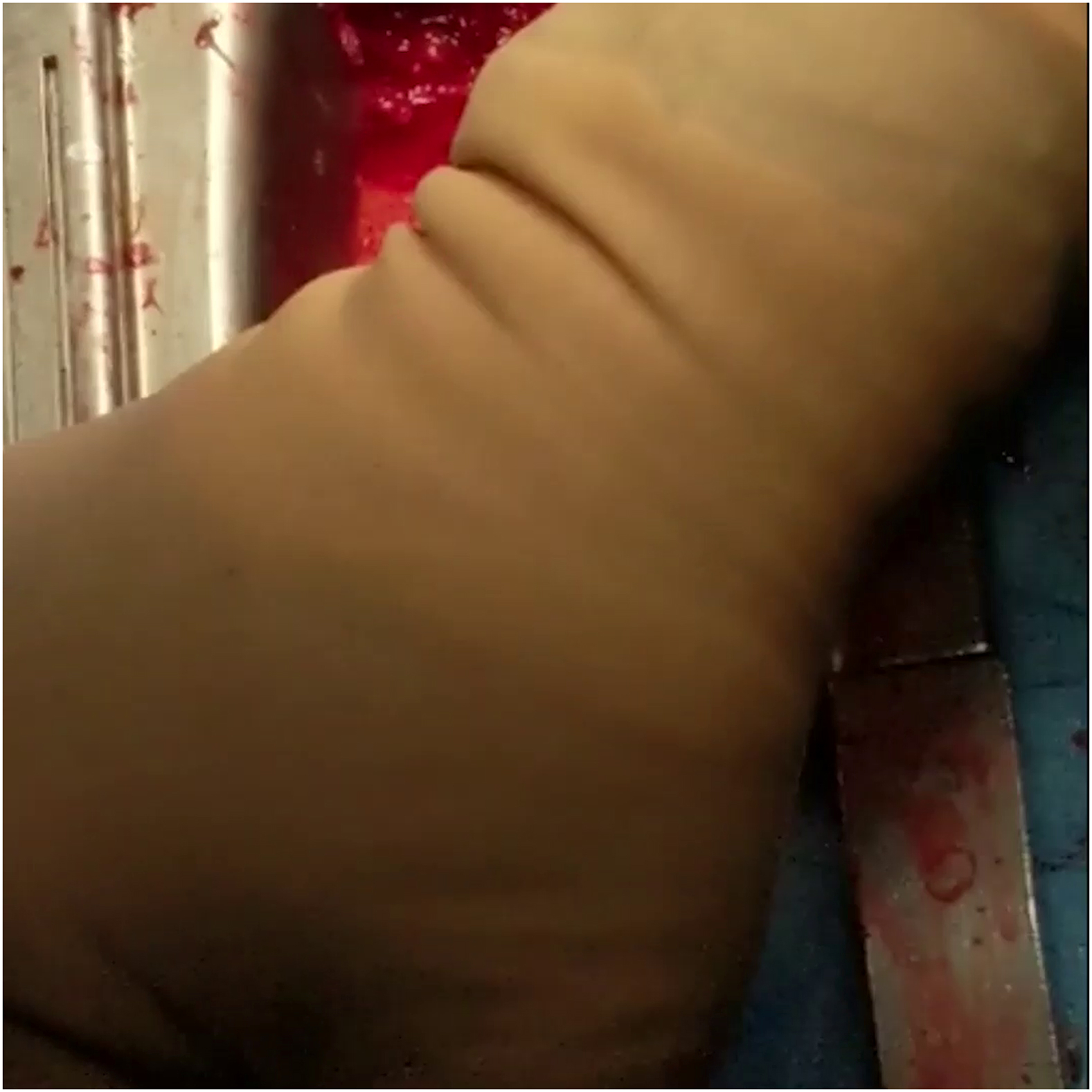

Supplement: Video 11 — Intraoperative view #11. Video available at: https://www.jtcvs.org/article/S2666-2736(26)00139-7/fulltext. [file fx12.jpg]

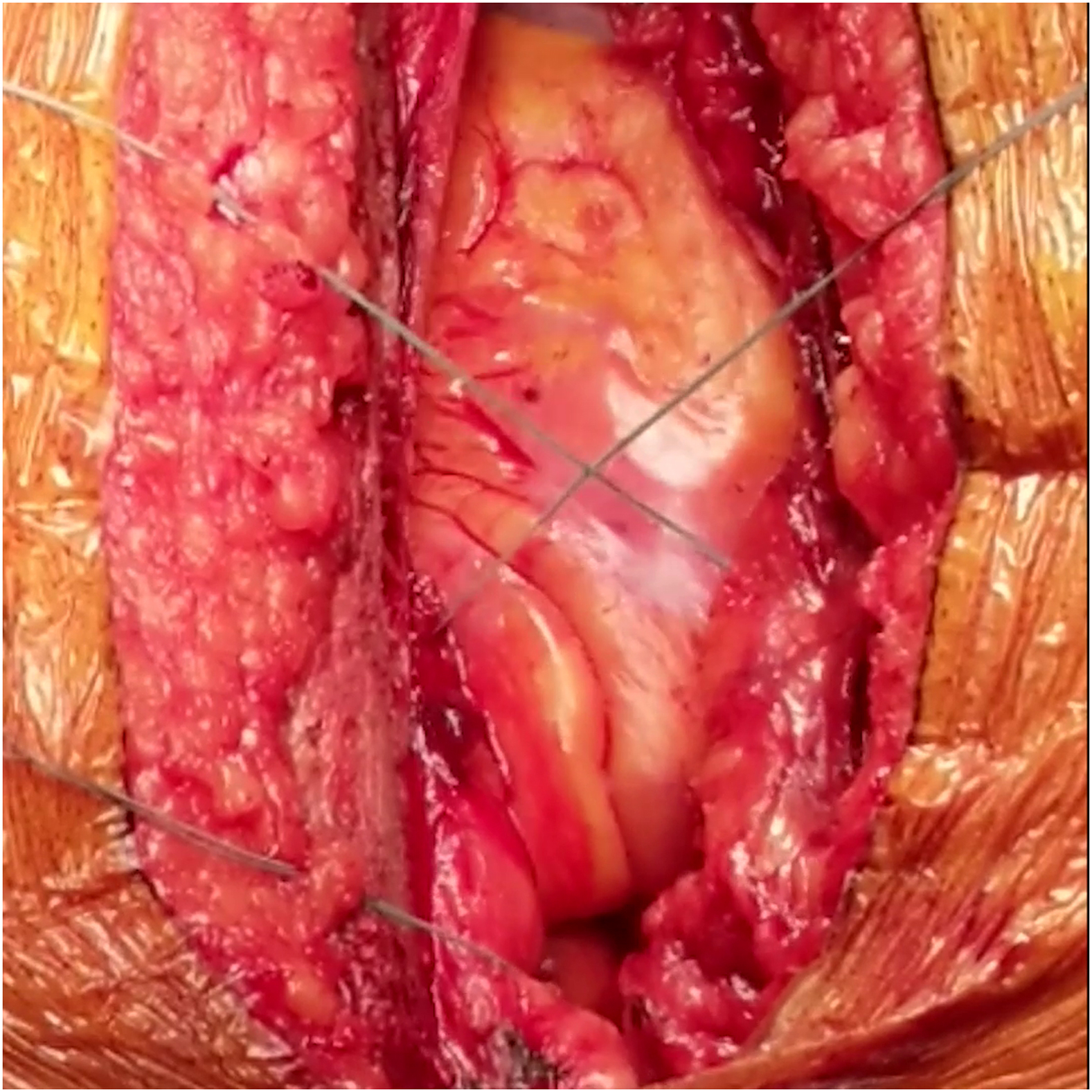

Supplement: Video 12 — Intraoperative view #12. Video available at: https://www.jtcvs.org/article/S2666-2736(26)00139-7/fulltext. [file fx13.jpg]

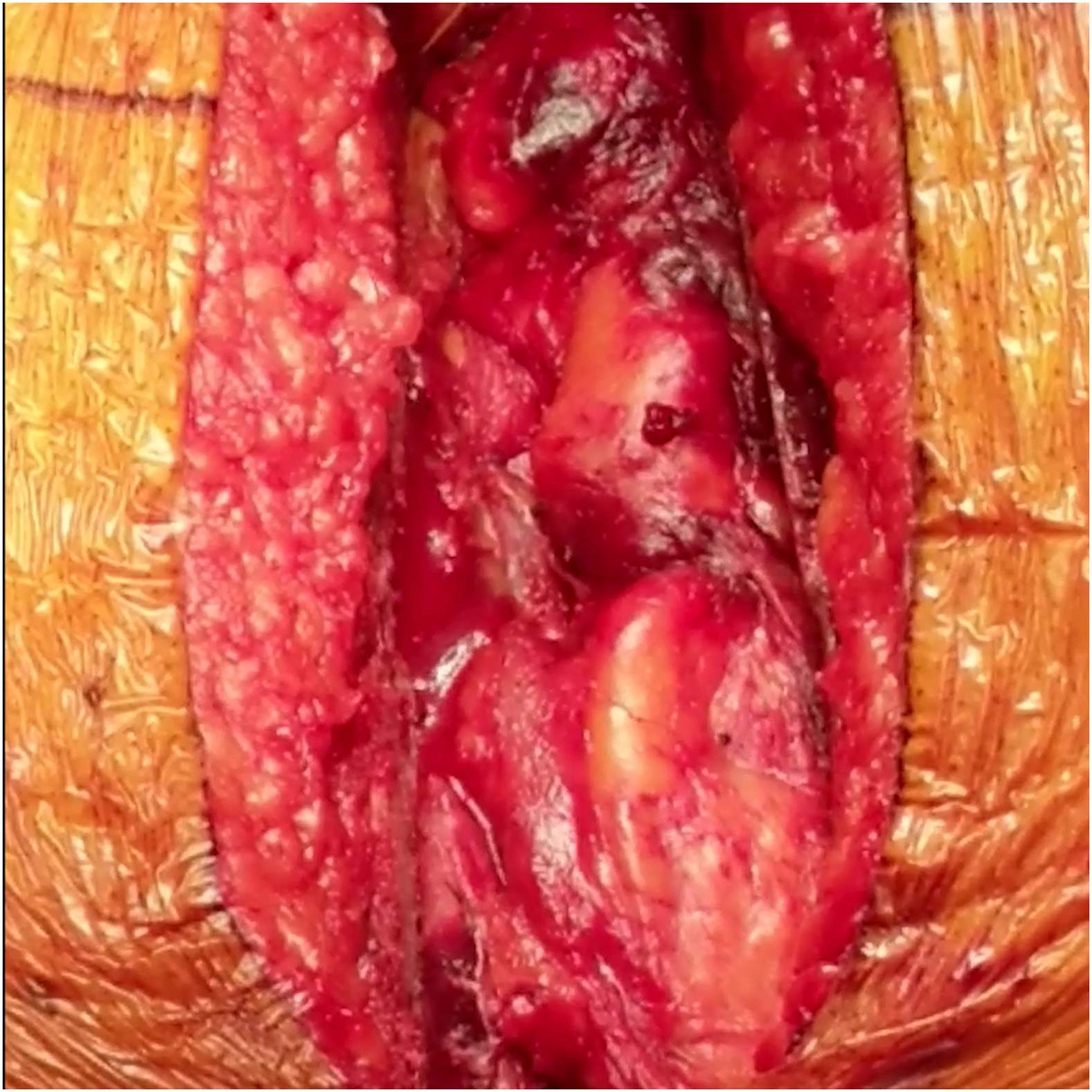

Supplement: Video 13 — Intraoperative view #13. Video available at: https://www.jtcvs.org/article/S2666-2736(26)00139-7/fulltext. [file fx14.jpg]

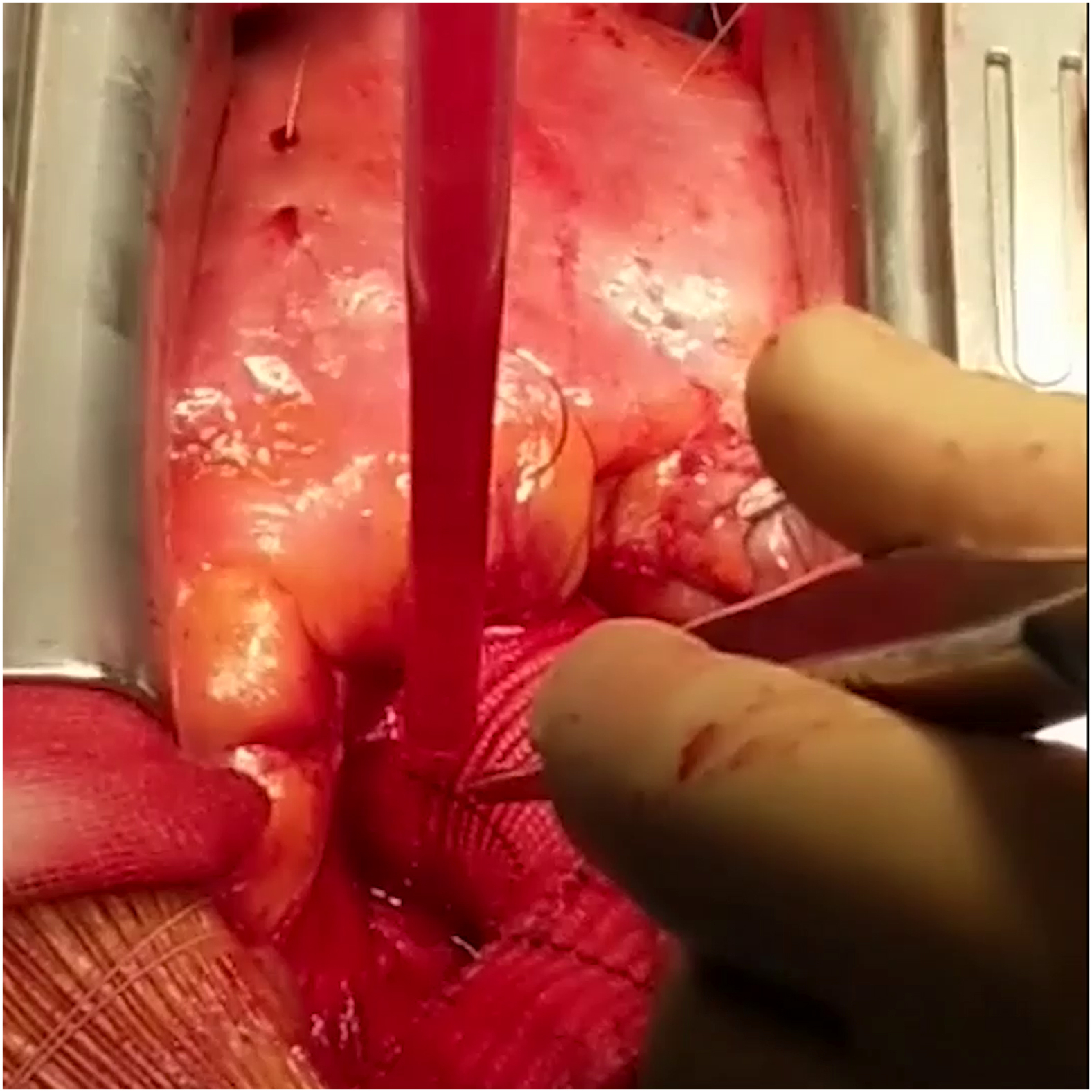

Supplement: Video 14 — Intraoperative view #14. Video available at: https://www.jtcvs.org/article/S2666-2736(26)00139-7/fulltext. [file fx15.jpg]

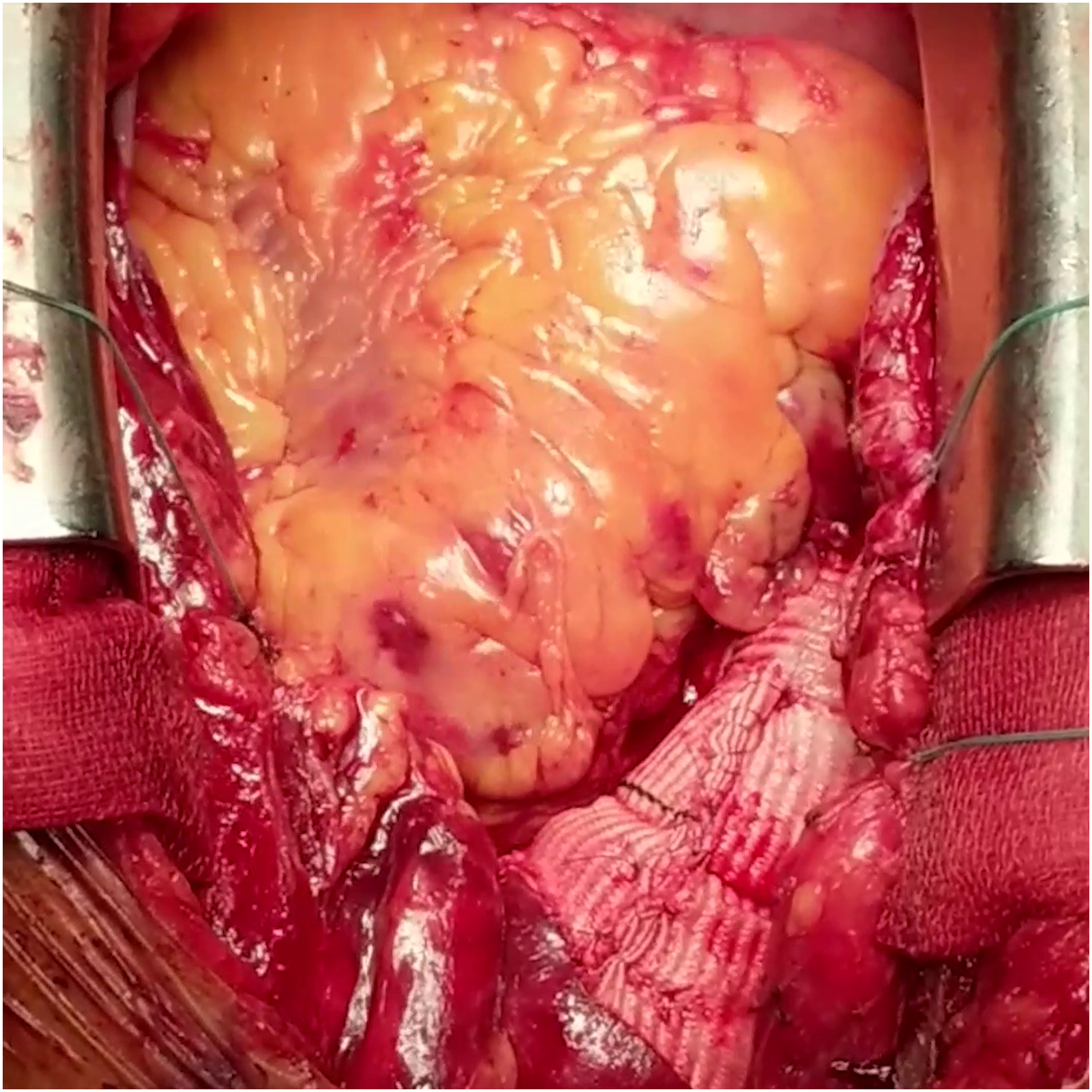

Supplement: Video 15 — Intraoperative view #15. Video available at: https://www.jtcvs.org/article/S2666-2736(26)00139-7/fulltext. [file fx16.jpg]

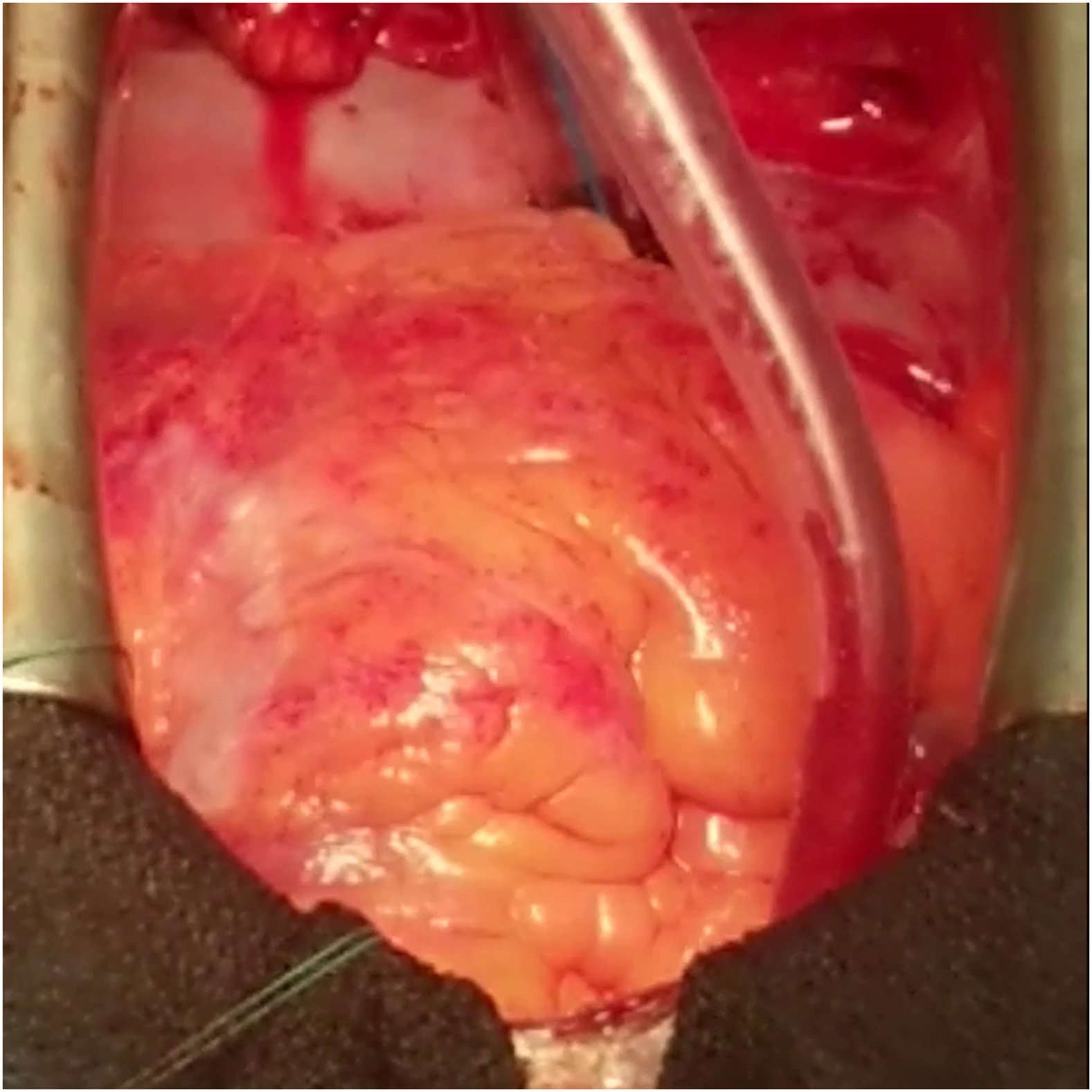

Supplement: Video 16 — Intraoperative view #16. Video available at: https://www.jtcvs.org/article/S2666-2736(26)00139-7/fulltext. [file fx17.jpg]

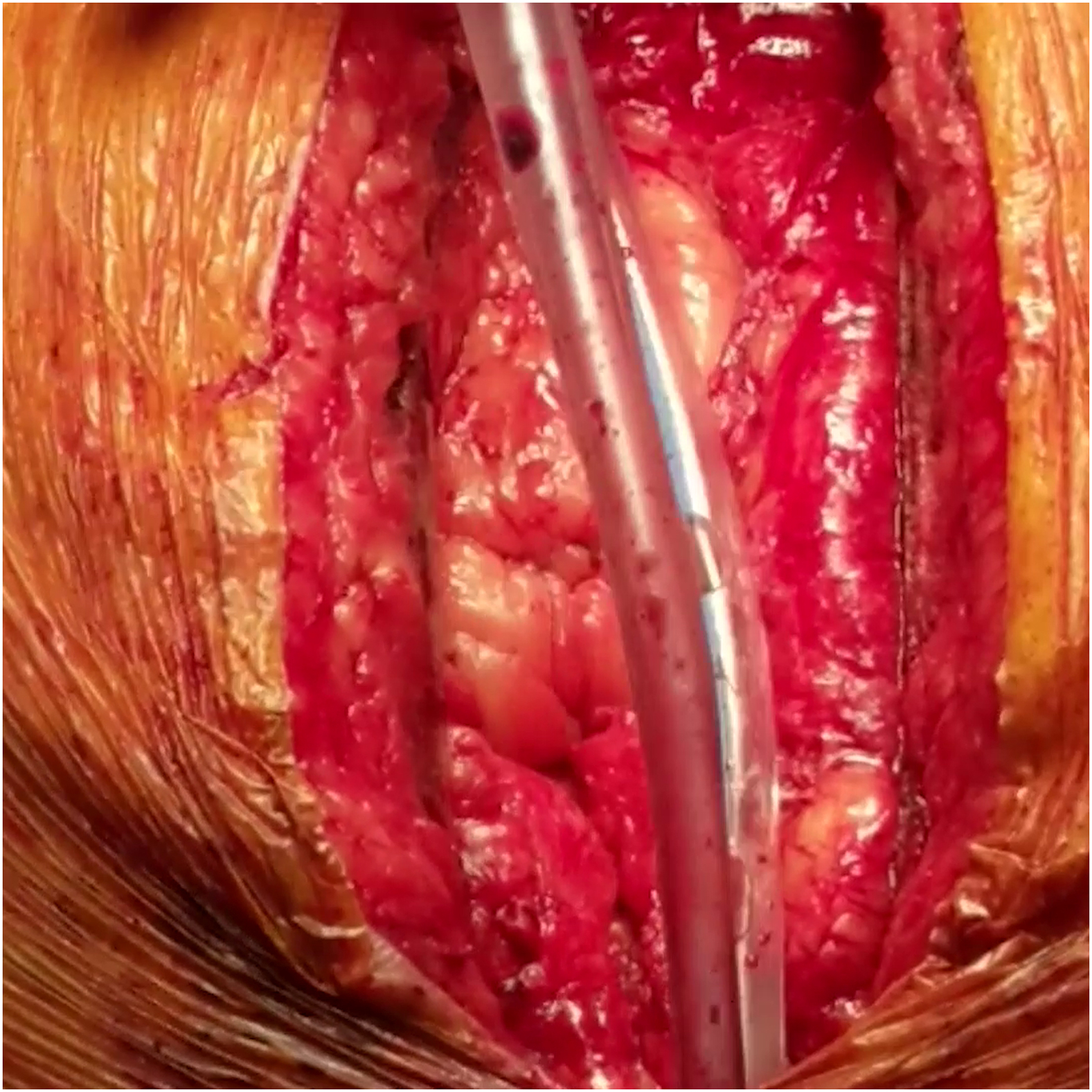

Supplement: Video 17 — Intraoperative view #17. Video available at: https://www.jtcvs.org/article/S2666-2736(26)00139-7/fulltext. [file fx18.jpg]
